# Exploring the Retinal Binding Cavity of Archaerhodopsin-3 by Replacing the Retinal Chromophore With a Dimethyl Phenylated Derivative

**DOI:** 10.3389/fmolb.2021.794948

**Published:** 2021-12-20

**Authors:** Taichi Tsuneishi, Masataka Takahashi, Masaki Tsujimura, Keiichi Kojima, Hiroshi Ishikita, Yasuo Takeuchi, Yuki Sudo

**Affiliations:** ^1^ Laboratory of Biophysical Chemistry, Graduate School of Medicine, Dentistry and Pharmaceutical Sciences, Okayama University, Okayama, Japan; ^2^ Laboratory of Synthetic and Medicinal Chemistry, Graduate School of Medicine, Dentistry and Pharmaceutical Sciences, Okayama University, Okayama, Japan; ^3^ Department of Applied Chemistry, The University of Tokyo, Tokyo, Japan; ^4^ Research Center for Advanced Science and Technology, The University of Tokyo, Tokyo, Japan

**Keywords:** retinal, rhodopsin, proton pump, derivative, photoreceptor

## Abstract

Rhodopsins act as photoreceptors with their chromophore retinal (vitamin-A aldehyde) and they regulate light-dependent biological functions. Archaerhodopsin-3 (AR3) is an outward proton pump that has been widely utilized as a tool for optogenetics, a method for controlling cellular activity by light. To characterize the retinal binding cavity of AR3, we synthesized a dimethyl phenylated retinal derivative, (2E,4E,6E,8E)-9-(2,6-Dimethylphenyl)-3,7-dimethylnona-2,4,6,8-tetraenal (DMP-retinal). QM/MM calculations suggested that DMP-retinal can be incorporated into the opsin of AR3 (archaeopsin-3, AO3). Thus, we introduced DMP-retinal into AO3 to obtain the non-natural holoprotein (AO3-DMP) and compared some molecular properties with those of AO3 with the natural A1-retinal (AO3-A1) or AR3. Light-induced pH change measurements revealed that AO3-DMP maintained slow outward proton pumping. Noteworthy, AO3-DMP had several significant changes in its molecular properties compared with AO3-A1 as follows; 1) spectroscopic measurements revealed that the absorption maximum was shifted from 556 to 508 nm and QM/MM calculations showed that the blue-shift was due to the significant increase in the HOMO-LUMO energy gap of the chromophore with the contribution of some residues around the chromophore, 2) time-resolved spectroscopic measurements revealed the photocycling rate was significantly decreased, and 3) kinetical spectroscopic measurements revealed the sensitivity of the chromophore binding Schiff base to attack by hydroxylamine was significantly increased. The QM/MM calculations show that a cavity space is present at the aromatic ring moiety in the AO3-DMP structure whereas it is absent at the corresponding *β*-ionone ring moiety in the AO3-A1 structure. We discuss these alterations of the difference in interaction between the natural A1-retinal and the DMP-retinal with binding cavity residues.

## Introduction

**FIGURE 1:**
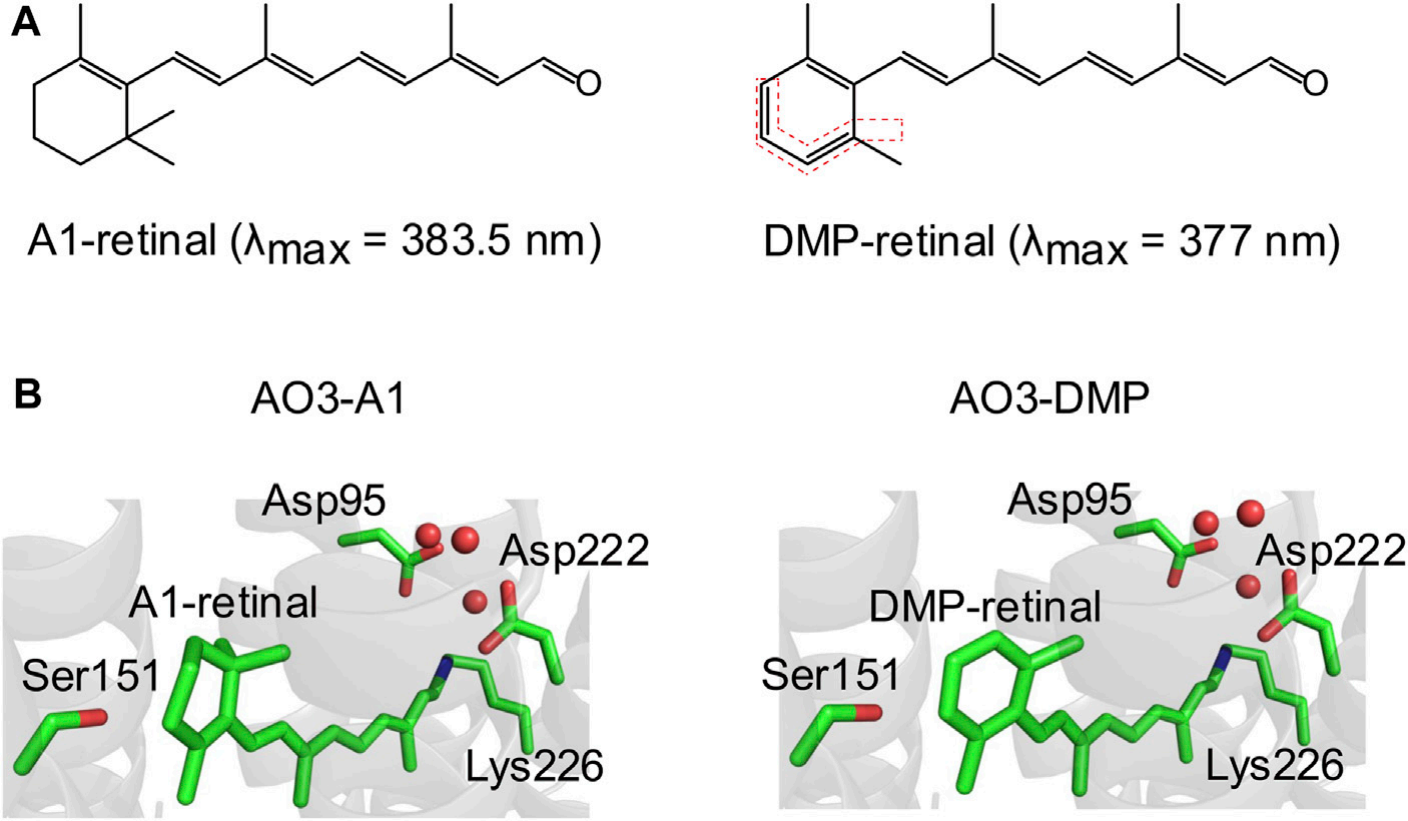
Structures of A1-retinal and its synthesized dimethyl phenylated retinal derivative (DMP-retinal). **(A)** Chemical structures of the natural chromophore all-*trans* A1-retinal **(left)** and its dimethyl phenylated derivative (DMP-retinal) **(right)**. The bonds highlighted by the red dotted lines indicate the modified groups. The absorption maxima (λ_max_) of A1-retinal and DMP-retinal in ethanol are shown in parentheses. **(B)** The QM/MM optimized structural environment of AO3 around the chromophore, A1-retinal **(left)** and DMP-retinal **(right)**. Red circles indicate water molecules.

Sunlight is utilized as an essential energy source and functions to provide significant external signals in many organisms, where photoreceptive proteins are responsible for the light reception. Rhodopsins are photoreceptor proteins that consist of seven-transmembrane α -helices and are widely distributed in all domains of life, archaea, bacteria and eukaryotes (Ernst et al., 2014; Govorunova et al., 2017). Rhodopsins consist of an apoprotein opsin and vitamin-A aldehyde retinal with A1-retinal being the natural chromophore (Figure 1A) (Shichida and Matsuyama, 2009; Takayama et al., 2018). Among the retinal isomers (e.g., 9-*cis*, 11-*cis* and 13-*cis*), all-*trans* retinal (A1-retinal) is the most thermally stable isomer and is utilized for rhodopsins expressed by microbes (hereafter microbial rhodopsins). A1-retinal binds covalently to a conserved Lys residue on the seventh (or G) helix of the opsin via a protonated retinal Schiff base (PRSB) linkage, where the positive charge is stabilized by a negatively charged carboxylate called the counterion (Figure 1B) (Ernst et al., 2014; Govorunova et al., 2017). Light absorption by rhodopsin triggers isomerization of the retinal chromophore within several hundred femtoseconds and the stored energy in the excited state induces a stepwise reaction with structural changes of the opsin that lead to a variety of photobiological functions including photo-energy conversion and photo-signal transduction (Ernst et al., 2014; Govorunova et al., 2017).

In addition to their biological significance, microbial rhodopsins have been widely utilized as tools for optogenetics, a method to control cellular activity by light *in vivo* (Zhang et al., 2011). For instance, light-gated cation channelrhodopsin (CCR) and anion channelrhodopsin (ACR) are employed as a neural activator and a neural silencer, respectively, because of their membrane depolarization and hyperpolarization ability, respectively (Boyden et al., 2005; Govorunova et al., 2015). In addition, archaerhodopsin-3 (AR3), expressed by the halophilic archaeon *Halorubrum sodomense* (Genbank accession number; WP_092921078), is utilized as an optogenetics tool. AR3 absorbs around 570 nm light, and that photoabsorption results in the sequential appearance of various photointermediates (e.g., K, M, N, and O), followed by a return to the unphotolyzed form of the protein (Maclaurin et al., 2013; Inoue et al., 2015). During each reaction cycle, a proton is actively transported from the intracellular to the extracellular side through the protein moiety (Inoue et al., 2015; Takayama et al., 2018). The outward proton transport activity induces membrane hyperpolarization and therefore AR3 is also used as a neural silencer as is ACR (Chow et al., 2010; Sudo et al., 2013). In addition, AR3 is also used for voltage imaging of neurons (Kralj et al., 2011; Flytzanis et al., 2014; Kojima et al., 2020a). This is due to the membrane voltage-dependent fluorescence changes of a highly fluorescent photo-intermediate, the Q intermediate. The AR3-based voltage indicators have two important advantages as follows: 1) they can be expressed in targeted neurons using genetics, and 2) it is possible to directly visualize the absolute membrane voltage with high temporal (500 μs–40 ms) resolution even below the threshold (Kralj et al., 2011).

So far, several research groups have applied various retinal derivatives into AR3 to modify the molecular properties as neural silencers and voltage indicators (Sineshchekov et al., 2012; Azimihashemi et al., 2014; Herwig et al., 2017; Ganapathy et al., 2019). The previous studies mainly aimed to produce red-shifted and near-infrared AR3 pigments. In this study, we synthesized a new dimethyl phenylated retinal derivative (2E,4E,6E,8E)-9-(2,6-dimethylphenyl)-3,7-dimethylnona-2,4,6,8-tetraenal (named DMP-retinal), with the purpose of further exploring the protein environment lining the retinal binding cavity of AR3 (Figure 1A). After DMP-retinal was incorporated into the apoprotein of AR3 (archaeopsin-3, AO3), the functional and spectroscopic properties of the non-natural protein (AO3-DMP) were characterized and compared with those of the natural protein (i.e., AR3). Of note, spectroscopic measurements revealed that the absorption maximum of AO3-DMP was significantly blue-shifted (508 nm) compared with AR3 (556 nm). By using QM/MM calculations, this absorption change was explained by the difference of HOMO-LUMO energy gap between A1-retinal and DMP-retinal, and the difference of electrostatic interactions of the retinal Schiff base with some residues around the chromophore. From these results and QM/MM calculations, the implications of DMP-retinal are discussed.

## Materials and Methods

### Synthesis of a Dimethyl Phenylated Retinal Derivative

A retinal derivative, DMP-retinal, which possesses a dimethyl phenylated group (Figure 1A), was newly synthesized by an organic chemistry method with overall yield including the *trans*- and *cis*-forms of 17% from 2,6-dimethylbenzaldehyde as a versatile synthon (Supplementary Figures S1, S2). UV-visible absorption spectra of all-*trans* A1-retinal and DMP-retinal in ethanol were recorded in the dark at room temperature using a UV-2600 spectrophotometer (Shimadzu Corp., Japan) to estimate their absorption maxima.

### Theoretical Calculations

The atomic coordinates of AR3 were taken from its X-ray structure (PDB code 6GUX (Bada Juarez et al., 2021)). All crystal water molecules and ions were included explicitly in calculations if not otherwise specified. During the optimization of hydrogen atom positions with CHARMM (Brooks et al., 1983), the positions of all heavy atoms were fixed and all titratable groups (e.g., acidic and basic groups) were ionized. The Schiff base was considered to be protonated. Atomic partial charges of the amino acids and retinal were obtained from the CHARMM22 (Mackerell et al., 1998) parameter set.

The computation of the protonation pattern was based on the electrostatic continuum model, solving the linear Poisson-Boltzmann equation with the MEAD program (Bashford and Karplus, 1990). The difference in electrostatic energy between the two protonation states, protonated and deprotonated, in a reference model system was calculated using a known experimentally measured p*K*_a_ value (e.g., 4.0 for Asp (Nozaki and Tanford, 1967)). The difference in the p*K*_a_ value of the protein relative to the reference system was added to the known reference p*K*_a_ value. The experimentally measured p*K*_a_ values employed as references were 12.0 for Arg, 4.0 for Asp, 9.5 for Cys, 4.4 for Glu, 10.4 for Lys, 9.6 for Tyr (Nozaki and Tanford, 1967), and 7.0 and 6.6 for the N_ε_ and N_δ_ atoms of His, respectively (Tanokura, 1983a; b; c). All other titratable sites were fully equilibrated to the protonation state of the target site during titration. The dielectric constants were 4 for the protein interior and 80 for water. All water molecules were considered implicitly. All computations were performed at 300 K, pH 7.0, and with an ionic strength of 100 mM. The linear Poisson-Boltzmann equation was solved using a three-step grid-focusing procedure at resolutions of 2.5, 1.0 and 0.3 Å. The ensemble of protonation patterns was sampled using the Monte Carlo (MC) method with the Karlsberg program (Rabenstein and Knapp, 2001). The MC sampling yielded the probabilities [protonated] and [deprotonated] of the two protonation states of the molecule. Next, the hydrogen atom positions were re-optimized with CHARMM (Brooks et al., 1983) in the calculated protonation states of the titratable residues. Using the resulting coordinates, the protonation state of the titratable residues was finally determined using the MEAD (Bashford and Karplus, 1990) and Karlsberg (Rabenstein and Knapp, 2001) programs.

#### In Protein

The geometry was optimized using a QM/MM approach. The restricted density functional theory (DFT) method was employed with the B3LYP functional and LACVP* basis sets using the QSite (Qsite, 2012) program. The QM region was defined as the all-*trans* A1/DMP-retinal Schiff base (including the Lys226 side chain), side chains within the van der Waals contact distance of the retinal Schiff base (Arg92, Tyr93, Asp95, Trp96, Thr99, Thr100, Leu103, Met128, Ile129, Trp148, Ser151, Thr152, Met155, Trp192, Tyr195, Trp199, Asp222, and Ala225) and water molecules near the Schiff base (H_2_O-401, 402, and 406). All atomic coordinates were fully relaxed in the QM region. In the MM region, the positions of H atoms were optimized using the OPLS2005 force field (Jorgensen et al., 1996), while the positions of the heavy atoms were fixed. The absorption wavelengths of AO3-A1 and AO3-DMP were calculated using the QM/MM-optimized structures (SI coordinates). A QM/MM approach with the polarizable continuum model (PCM) method with a dielectric constant of 78 for the bulk region, in which electrostatic and steric effects created by a protein environment were explicitly considered in the presence of bulk water, was employed (QM/MM/PCM approach). In the PCM method, the polarization points were placed on spheres with a radius of 2.8 Å from the center of each atom in order to model possible water molecules in the cavity. Radii of 2.8–3.0 Å from each atom center and the dielectric constant value of ∼80 are likely to be optimal to reproduce the excitation energetics, as evaluated for the QM/MM/PCM approach (Tamura et al., 2020). The QM/MM/PCM approach with the B3LYP functional and 6-31G* basis sets was employed using the GAMESS program (Schmidt et al., 1993). The protonation pattern of the titratable residues was implemented in the atomic partial charges of the MM region. The atomic partial charges of the other residues in the MM region were obtained from the CHARMM22 (Mackerell et al., 1998) parameter set. The absorption energy of microbial rhodopsins is highly correlated with the energy difference between the highest occupied molecular orbital (HOMO) and the lowest unoccupied molecular orbital (LUMO) of the retinal Schiff base (Δ*E*_HOMO-LUMO_) (Tsujimura and Ishikita, 2020; Tsujimura and Ishikita, 2021; Tsujimura et al., 2021). To calculate the absorption energies and corresponding wavelengths, the QM region was redefined to only include the retinal Schiff base, and the energy levels of the HOMO and LUMO were calculated. The absorption energy (*E*_abs_ in eV) was calculated using the following equation (obtained for 13 microbial rhodopsins; coefficient of determination *R* ^2^ = 0.995) (Tsujimura and Ishikita, 2020):

$$E_{\mathrm{abs}}=1.360\;\Delta E_{\mathrm{HOMO}-\mathrm{LUMO}}-1.018$$ (1)

The electrostatic contribution of the side chain in the MM region to the absorption wavelength of the retinal Schiff base was obtained consistently using the QM/MM/PCM approach.

#### In Vacuum

To calculate the absorption energy of the retinal Schiff base in vacuum (i.e., in the absence of the protein environment), the HOMO−LUMO energy gap of the retinal Schiff base in vacuum was calculated with the B3LYP/LACVP* function using the Jaguar program (Jaguar, 2012). The calculated HOMO-LUMO energy gap was corrected to the absorption energy using Eq. 1.

### Production and Purification of AO3-A1 and AO3-DMP

The expression plasmid for histidine-tagged AO3 was constructed as described previously (Takayama et al., 2018; Kojima et al., 2020b). The procedure for protein expression was essentially the same as previously described (Takayama et al., 2018). In short, *E. coli* BL21 (DE3) cells harboring the plasmid were grown at 37°C in LB medium supplemented with ampicillin (Wako Pure Chemical Industries, Ltd., Japan; final concentration of 50 μg/ml). When the optical density at 660 nm reached ca. 1.4–1.7, _L_-arabinose (Wako Pure Chemical Industries, Ltd., Japan; final concentration of 0.1%) was added into the medium to induce expression of the apoprotein. Simultaneously, each chromophore (all-*trans* A1-retinal or DMP-retinal) was added to the medium (final concentration of 10 µM). After induction for 3 h at 37°C, *E. coli* cells were harvested by centrifugation (4,000 × g for 10 min) at 4°C. For estimation of the total amounts of photoactive proteins by spectroscopic measurements, the cells were disrupted by sonication according to our previous study (Kojima et al., 2020b). Absorption spectra of the suspensions of cell membranes were measured using a UV-2450 spectrophotometer (Shimadzu Corp., Japan) with an ISR2200 integrating sphere (Shimadzu, Japan) at room temperature (approx. 28°C). The absorption spectra were mathematically deconvoluted to estimate the total amounts of photoactive holoproteins according to our previous study (Kojima et al., 2020b). From the absorbance at 558 (AO3-A1) and 494 nm (AO3-DMP) with the molecular coefficient of AO3-A1 (45,000 cm^−1^ M^−1^) (Kojima et al., 2020a), we roughly calculated the protein amounts.

For purification of AO3-A1 and AO3-DMP, the harvested *E. coli* cells were suspended in 50 mM Tris-HCl (pH 7.0) buffer containing 300 mM NaCl. The cells were cooled in ice-cold water and then disrupted by sonication (UD-211, TOMY Seiko Co., Ltd., Japan; Output 7, Duty 50 (1 pulse/0.5 s), total time of 30 min). The membrane fraction was collected by ultracentrifugation (himac CP56G, Hitachi Koki, P50A2 rotor, 4°C, 134,189 × g, 60 min) and then homogenized in the same buffer. The detergent *n*-dodecyl-β-D-maltoside (DDM, Dojindo Laboratories, Japan) was added to the suspension at a final concentration of 1.0% (w/v) to solubilize *E. coli* membranes containing AO3 with all-*trans* A1-retinal or DMP-retinal. After another ultracentrifugation step, the supernatant was passed through a HisTrap FF prepacked column (GE Healthcare, USA) to adsorb the C-terminal histidine-tagged protein for purification. The protein bound on the resin was washed with 50 mM Tris-HCl (pH 8.0) buffer containing 1 M NaCl, 0.1% DDM and 20 mM imidazole, and was then eluted by gradually increasing the concentration of imidazole in the ÄKTA purifier chromatography system (GE Healthcare, USA) at 4°C. The purified proteins were concentrated by centrifugation (4,000 × g, 4°C) using an Amicon Ultra Filter (30,000 MW cutoff; Merck Millipore, USA). The sample medium was exchanged with the 50 mM Tris-HCl (pH 7.0) buffer containing 300 mM NaCl, 0.05% DDM by centrifugation for more than 3 times.

### Light-Driven Outward Proton Transport Measurements

Proton transport activities were measured by light-induced pH changes of the cell suspension (Takayama et al., 2018; Kojima et al., 2020b). *E. coli* cells expressing AO3 with A1-retinal or DMP-retinal were washed three times in deionized water containing 300 mM NaCl and were finally suspended in the same solution. pH changes were monitored using a LAQUA F-72 pH meter equipped with a micro pH electrode (Horiba, Ltd., Japan). Each cell suspension was kept in the dark for several minutes and then was illuminated with a 300 W Xe light source MAX-303 (Asahi Spectra Co., Ltd., Japan) equipped with a Y44 cut-off filter (>420 nm) for 3 min. Carbonyl cyanide 3-chlorophenylhydrazone (CCCP, Sigma-Aldrich, final concentration of 40 μM) was used as a proton-selective ionophore. The initial slope amplitudes of the light-induced pH changes from 0 to 10 s for AO3-A1 and 20–40 s for AO3-DMP after light irradiation were used as the index of proton pumping activity (Kojima et al., 2020b).

### Spectroscopic Measurements and Data Analysis

UV-visible absorption spectra were recorded at room temperature using a UV-2450 spectrophotometer (Shimadzu Corp., Japan). The purified samples were solubilized in 50 mM Tris-HCl (pH 7.0) buffer containing 300 mM NaCl and 0.05% DDM. For flash-photolysis experiments, the membrane fractions were suspended in 50 mM Tris-HCl (pH 7.0) buffer containing 300 mM NaCl. The temperature was kept at 25°C using a thermostat. The experiments were carried out using a homemade computer-controlled flash-photolysis apparatus equipped with an Nd:YAG laser (Surelite I-10, Continuum, USA) and optical components as described previously (Inoue et al., 2018). The wavelength of the actinic pulse was tuned at 555 nm (AO3-A1) and at 505 nm (AO3-DMP). The pulse width and intensity were adjusted to 4 ns and 2 mJ/pulse, respectively. Photo-induced absorption changes were measured at each wavelength from 390 to 730 nm. 30 temporal traces were averaged at each wavelength to improve the signal-to-noise ratio. Data taken before the flash of light were adopted as a baseline. After measurement, we analyzed the time-dependent absorption changes of each wavelength at 410 nm (AO3-A1) and at 390 nm (AO3-DMP) for the M intermediate, at 550 nm (AO3-A1) and at 480 nm (AO3-DMP) for the original state, and at 640 nm (AO3-DMP) and at 580 nm (AO3-DMP) for the O intermediate. To analyze the data, we used the following equation for each state;

$$F\left(x\right)=A_{1}\exp\left(-\frac{x}{t_{1}}\right)+A_{2}\exp\left(-\frac{x}{t_{2}}\right)+A_{3}\exp\left(-\frac{x}{t_{3}}\right)$$

where *t* represents each time constant and *A* represents the rate of each intermediate at each time constant. After the measurements, the reproducibility of the data was checked to confirm that the sample was not denatured during the measurements.

### Reaction With Hydroxylamine

For the reaction with hydroxylamine, *E. coli* membranes expressing AO3-A1 or AO3-DMP were prepared according to the previous study (Kojima et al., 2020b). The samples were suspended in 50 mM Tris-HCl (pH 7.0) buffer containing 300 mM NaCl supplemented with 100 mM hydroxylamine at room temperature (approx. 28°C) in the dark. The bleaching processes were monitored using a UV-2450 spectrophotometer with an ISR2200 integrating sphere (Shimadzu, Japan).

## Results and Discussion

### Synthesis of DMP-Retinal and Model Structures for AO3 With A1-Retinal and DMP-Retinal

A retinal derivative possessing a dimethyl phenylated group, DMP-retinal (Figure 1A), was newly synthesized by an organic chemistry method with an overall yield of 17% from 2,6-dimethylbenzaldehyde as a versatile synthon (Supplementary Figures S1, S2). In ethanol, DMP-retinal was yellow in color (λ_max_ = 377 nm) as was natural all-*trans* A1-retinal (λ_max_ = 383.5 nm). The slight spectral blue-shift (6.5 nm) cannot be explained by the extension of the π-conjugation system on the polyene chain of the chromophore.

Many kinds of retinal derivatives have been synthesized and their effects on microbial rhodopsins have been investigated (Sineshchekov et al., 2012; Azimihashemi et al., 2014; Herwig et al., 2017; Ganapathy et al., 2019). Although (2E,4E,6E,8E)-9-phenyl-3,7-dimethylnona-2,4,6,8-tetraenal (named PHE) and (2E,4E,6E,8E)-9-(2,6-Dimethyl-4-methylamino)phenyl-3,7-dimethylnona-2,4,6,8-tetraenal (named MMAR) are closely related to DMP-retinal from the chemical aspects (Figure 1; Supplementary Figure S3), MMAR can be incorporated into AO3 but PHE cannot (Ganapathy et al., 2019). On the other hand, PHE can be into the apoprotein of bacteriorhodopsin which shows high sequence identity (57%) with AR3 (Derguini et al., 1984). Thus, a common rule to evaluate whether retinal derivatives can be incorporated into the apoproteins of rhodopsins remains unclear. To examine whether DMP-retinal is incorporated into AO3 like the natural chromophore (i.e., all-*trans* A1-retinal), we performed QM/MM calculations (see *Materials and Methods*). The model structure of AO3 with DMP-retinal suggested that the chromophore surrounding residues such as Asp95, Ser151, Asp222 and Lys226 are not altered upon DMP binding (Figure 1B). In addition, the water cluster (red circles) around the Schiff base was conserved in AO3-DMP (Figure 1B). Thus, we estimated that DMP-retinal could be incorporated into the retinal binding pocket of AO3 (Figure 1B).

### Production of AO3 With DMP-Retinal and Its Proton Transport Activity

**FIGURE 2:**
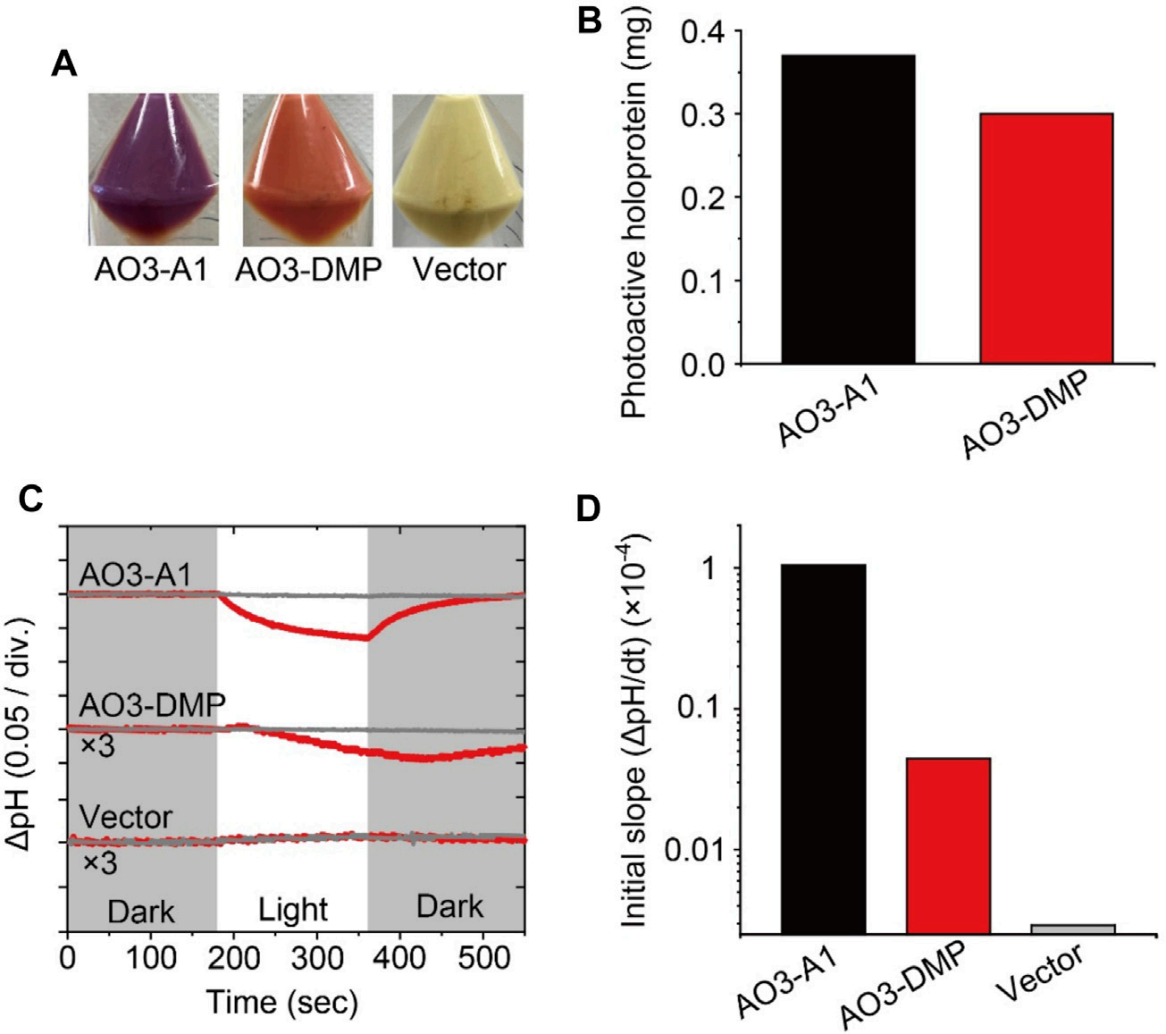
Proton transport activity of AO3 with A1-retinal and DMP-retinal. **(A)** Photographs of *E. coli* cells harboring the expression plasmid for AO3 in the presence of all-*trans* A1-retinal **(AO3-A1, left)** and DMP-retinal **(AO3-DMP, middle)**. A photograph of the plasmid vector **(Vector, right)** is shown as a negative control. **(B)** Comparison of the total amounts of photoactive holoproteins in *E. coli* cells expressing AO3-A1 and AO3-DMP. **(C)** Light-induced pH changes of *E. coli* cells harboring expression plasmids of AO3 with A1-retinal (AO3-A1) and DMP-retinal (AO3-DMP) in the presence (gray lines) or absence (red lines) of the proton-selective ionophore, CCCP (final concentration of 4 μM). The amplitudes of pH changes for AO3-DMP and the Vector were enlarged 3 times for comparison. The initial pH ranged from 6.1 to 6.3. The white background indicates the period of illumination. **(D)** The initial slope amplitudes of the light-induced pH changes, which were obtained from the data in panel C.

To experimentally determine whether DMP-retinal is incorporated into AO3, we first expressed the opsin of AR3 (archaeopsin-3, AO3) in the presence of natural all-*trans* A1-retinal (A1-retinal) or DMP-retinal in *E. coli* cells. The cells showed purple and orange colors for AO3 with A1-retinal (AO3-A1) and DMP-retinal (AO3-DMP), respectively (Figure 2A), while the vector control showed a brown color originating from the *E. coli* cells. These results imply that DMP-retinal was successfully incorporated into AO3 as was A1-retinal via the protonated Schiff base linkage. The changes in color suggest that the absorption maximum of AO3-DMP is blue-shifted compared with AO3-A1. DMP-retinal is closely related to two retinal derivatives, PHE and MMAR (Supplementary Figure S3), which have been applied to AR3 (Ganapathy et al., 2019). Compared with DMP-retinal, methyl groups on the phenyl ring are absent in PHE and a methylamino group is added to the phenyl ring in MMAR (Derguini et al., 1984; Ganapathy et al., 2017; Ganapathy et al., 2019). PHE was shown not to generate visible light-absorbing pigment, which suggests the methyl groups of DMP-retinal play important roles to be incorporated into the retinal binding pocket as well as A1-retinal probably due to the interaction with amino acid residues of AO3 (e.g., Ile129, Ser151 and Met155). Indeed, the ligand binding energy calculation shows that the two methyl groups in DMP-retinal contribute to increases in van der Waals contact with Trp96 (3.3 Å), Trp199 (3.6 Å), Pro196 (3.7 Å), and Ser151 (3.3 Å) (Supplementary Figure S4). On the other hand, MMAR generated red-shifted pigment in comparison with AO3-A1 (Ganapathy et al., 2019). The red-shifted effects were also observed in mutants of AO3 and other microbial rhodopsins, such as proteorhodopsin and Gloeobacter rhodopsin (Ganapathy et al., 2017; Mei et al., 2020). The methylamino group at the phenyl ring in MMAR may affect the electronic structure of the retinal, probably leading to the red-shifted absorption wavelength. Noteworthy, in contrast to MMAR, DMP-retinal generated blue-shifted pigment.

To investigate the regeneration levels of A1-retinal and DMP-retinal on AO3, we estimated the total amounts of photoactive proteins by estimating the absorbance derived from holoproteins in *E. coli* cells according to our previous report (Supplementary Figure S5) (Kojima et al., 2020b). The total amount of AO3-A1 was only 1.2-fold larger than that of AO3-DMP (Figure 2B). Thus, regeneration level of AO3-DMP was similar with that of AO3-A1 in the membranes. Microbial opsins including AO3 probably contain a special entrance channel for the chromophore, which is located in the extracellular membrane segments (Isralewitz et al., 1997). We speculate that DMP-retinal exhibits similar permeability in the proposed channel and similar formation rate of the protonated Schiff base in the retinal binding pocket compared with A1-retinal, which leads to the comparable regeneration level. Judging from the similar regeneration level of AO3-DMP with that of AO3-A1, DMP-retinal would be adequately incorporated into AO3 in animal cells for optogenetic application.

Then, we investigated the function of AO3 with DMP-retinal. As shown in Figures 2A,C light-induced pH decrease was observed for cells expressing AO3-A1 and the pH change was impaired by adding the protonophore carbonyl cyanide 3-chlorophenylhydrazone (CCCP) (upper panel in Figure 2C), while the vector control showed no significant change (lower panel in Figure 2C) (Takayama et al., 2018). These results are consistent with previous studies and indicate that AO3-A1 works as an outward proton pump (Table 1) (Takayama et al., 2018). For AO3-DMP, a light-induced slight but significant pH decrease was observed, and the pH change was impaired by adding CCCP, indicating that AO3-DMP works as an outward proton pump as does AO3-A1. Then, to compare their proton pump activities, we obtained initial slope amplitudes of the light-induce pH decrease after illumination in the absence of CCCP as index of proton pumping activities (Figure 2D). The amplitude of AO3-DMP was 15-fold smaller than that of AO3-A1, which indicates that the proton pump activity of AO3-DMP was smaller than that of AO3-A1. Moreover, the recovery of pH changes for AO3-DMP was delayed with the turn-off of the light. As described below, we hypothesized that the lower proton pumping activity and delayed recovery of pH changes of AO3-DMP is due to its slow photocycle reaction.

**TABLE 1:** Functional and photochemical properties of AO3 with all-*trans* A1-retinal and DMP-retinal. Photocycling rates represent inverse numbers of τ_3_, the decay time constant of O intermediate, which is a rate limiting step of the photocycle.

Opsin with chromophore	Function	Absorption maximum (nm)	Bleaching rate by reaction with hydroxylamine (min^−1^)	Photocycling rate (ms^−1^)
AO3-A1	Proton pump	556	3.91 × 10^–4^	1.19 × 10^–2^
AO3-DMP	Proton pump	508	6.61 × 10^–3^	1.13 × 10^–3^

### Absorption Properties of AO3 With A1-Retinal and DMP-Retinal

**FIGURE 3:**
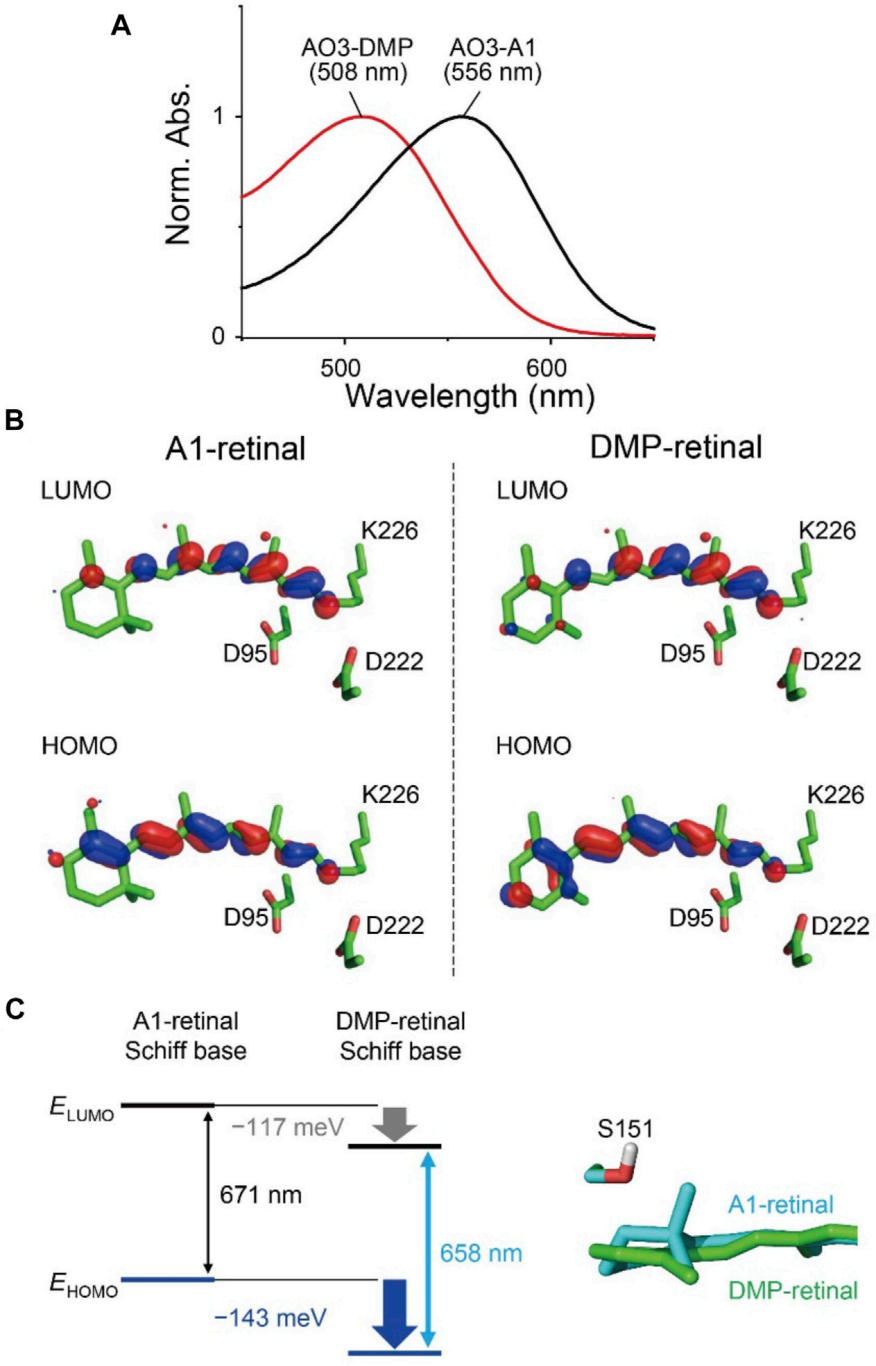
Absorption properties of AO3 with A1-retinal and DMP-retinal. **(A)** Absorption spectra of purified AO3 with A1-retinal (AO3-A1, black line) and DMP-retinal (AO3-DMP, red line). **(B)** HOMO and LUMO for A1-retinal **(left)** and DMP-retinal **(right)** Schiff bases in the AO3 protein environments. **(C)** Difference in HOMO and LUMO energy between A1-retinal and DMP-retinal Schiff bases in the absence of the protein environment (i.e. in vacuum) **(left)** and difference in the planarity between the *β*-ionone ring moiety in AO3-A1 (cyan) and the aromatic ring moiety in AO3-DMP (green) **(right)**. The data in panel **(C)** corresponds to the structure in panel B.

We investigated the absorption properties of AO3 with DMP-retinal (Figure 3). Firstly, we measured the absorption spectrum of purified AO3-DMP and compared it with AO3-A1 (Figure 3A). In spite of the potential extension of the π-conjugation system, the absorption maximum of AO3-DMP was significantly blue-shifted (508 nm) compared with AO3-A1 (556 nm) (Table 1). The spectral blue-shift (1,699 cm^−1^) is primarily explained by an increase in the energy gap between the electronic ground- and excited-states of the chromophore (Ernst et al., 2014; Tsujimura and Ishikita, 2020).

To elucidate factors that blue-shift the absorption wavelength of AO3-DMP, we performed QM/MM calculations. The calculated absorption wavelengths are 558 nm for AO3-A1 (i.e., AR3) and 519 nm for AO3-DMP (Table 2), which substantially reproduces the experimentally measured absorption wavelengths, 556 nm for AO3-A1 and 508 nm for AO3-DMP (Figure 3A). The calculated absorption wavelength of DMP-retinal Schiff base is 13 nm shorter than that of A1-retinal Schiff base in the absence of the protein environment (i.e. in vacuum) (Table 2). The extension of the *π*-conjugation system typically leads to an increase in the absorption wavelength due to the decrease of the HOMO-LUMO energy gap. In retinal, the HOMO and the LUMO are localized at the *β*-ionone ring moiety and the Schiff base moiety, respectively (Fujimoto et al., 2007) (Figure 3B). The HOMO energy level in DMP-retinal Schiff base is lower than that in A1-retinal Schiff base (143 meV), as the HOMO of DMP-retinal Schiff base is more delocalized over the entire molecule than that of A1-retinal Schiff base owing to the presence of the aromatic ring (Figures 3B,C). The corresponding stabilization of the LUMO level is small (117 meV), as the LUMO is less populated in both the aromatic and *β*-ionone ring moieties, but is more populated in the Schiff base moiety (Figures 3B,C). The decrease in the HOMO energy level with respect to the LUMO energy level in DMP retinal can partly explain why the substitution of A1-retinal with DMP-retinal blue-shifts the absorption wavelength. This can also explain why the absorption wavelength of DMP-retinal is 6.5 nm shorter than that of A1-retinal in ethanol (Figure 1; Supplementary Table S1), despite of the extended *π*-conjugation system in DMP-retinal.

**TABLE 2:** Calculated (calc.) and experimentally measured (expl.) absorption wavelengths for AO3-A1 and AO3-DMP (nm).

	Groups	AO3-A1	AO3-DMP	Difference
expl		556	508	48
calc. (in protein)		558	519	39
calc. (in vacuum)		671	658	13

Most of the remaining difference in the absorption wavelength is due to the difference in the electrostatic contributions of charged/polar residues near the retinal Schiff base, namely, the counterions Asp222 (67 meV) and Asp95 (56 meV), and Ser151 (15 meV) (Table 3; Supplementary Table S2). Negatively charged groups at the Schiff base moiety stabilize the S_0_ (ground) and S_1_ (lowest singlet excited) states of the retinal Schiff base (Tsujimura and Ishikita, 2020; Tsujimura et al., 2021). Because the Schiff base moiety is more positively charged in the S_0_ state than in the S_1_ state (Supplementary Figures S6A–C), counterions stabilize the S_0_ state with respect to the S_1_ state and decrease the absorption wavelength. In the S_1_ state, the aromatic/*β*-ionone ring region of the retinal Schiff base is more positively charged in the DMP-retinal Schiff base than in the A1-retinal Schiff base (Supplementary Figure S6B). Thus, in the AR3 protein environment, the stabilization of the S_1_ state owing to the electrostatic interactions with the counterions is not pronounced in the DMP-retinal Schiff base relative to the A1-retinal Schiff base (Supplementary Figure S6D). It should also be noted that the distances between the counterions and the retinal Schiff base do not differ between AO3-A1 and AO3-DMP (SI coordinates). On the other hand, Ser151 is closer to the *β-*ionone ring in AO3-A1 than to the aromatic ring in AO3-DMP due to the difference in the planarity of the ring moieties (O_Ser151_…C5_retinal_ distances are 3.16 Å in AO3-A1 and 3.34 Å in AO3-DMP) (Figure 3B). That difference makes the electrostatic interaction between Ser151 and DMP-retinal slightly weaker, which contributes to the increase in absorption energy of 15 meV in AO3-DMP with respect to AO3-A1 (Table 3; Supplementary Table S2).

**TABLE 3:** Contributions of the major residues to the absorption energies in AO3-A1 and AO3-DMP (meV).

Residues	AO3-A1	AO3-DMP	Difference
Asp222	174	241	67 (≈11 nm)
Asp95	207	263	56 (≈7 nm)
Ser151	−59	−44	15 (≈5 nm)

Thus, the absorption change was explained by two factors: 1) the difference of HOMO-LUMO energy levels between A1-retinal and DMP-retinal in the absence of the protein environment, and 2) the difference of electrostatic interactions of the Schiff base with three amino acids (Asp222, Asp95, Ser151). It should also be noted that there is a 9 nm gap between the experimentally measured absorption difference (48 nm) and the calculated absorption difference (39 nm). The slight difference may be due to the absence of structural fluctuation of the protein in the model structures, which is a source of the broadening of the absorption spectra.

### Photocycle Kinetics of AO3 With A1-Retinal and DMP-Retinal

**FIGURE 4:**
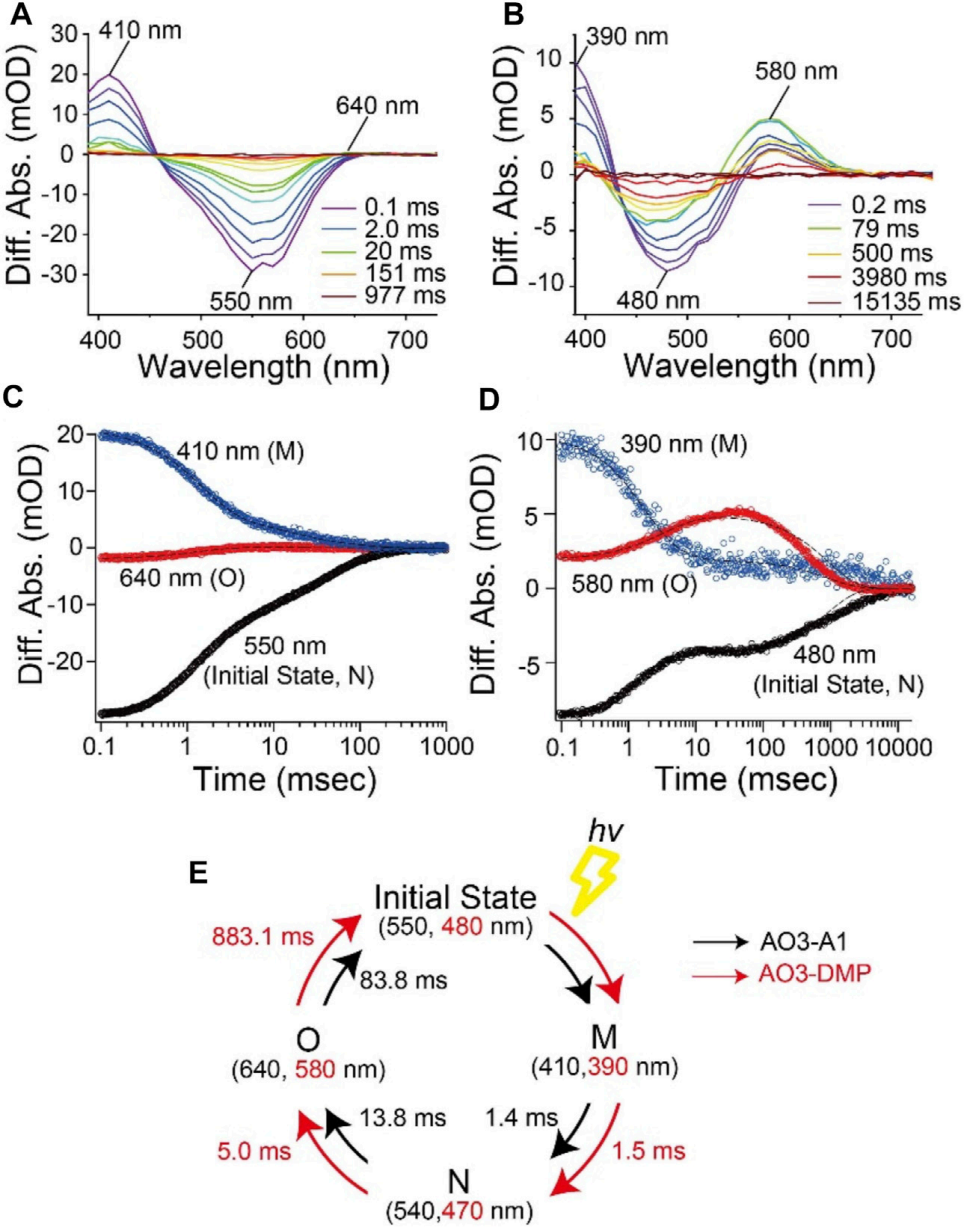
Photocycle kinetics of AO3 with A1-retinal and DMP-retinal. Flash-induced difference absorption spectra of purified AO3 with A1-retinal (AO3-A1) **(A)** and DMP-retinal (AO3-DMP) **(B)** over the spectral range of 390–730 nm. The time courses of absorbance changes of AO3-A1 at 410, 550 and 640 nm **(C)** and absorbance changes of AO3-DMP at 390, 480, and 580 nm **(D)**, where the black dotted lines show the fitting curves. **(E)** A model of the photocycle of AO3-A1 (black) and AO3-DMP (red). The absorption maxima and reaction rates are overlaid.

To investigate the photocycle kinetics, we carried out flash-photolysis experiments from millisecond to second time frames (Figure 4). *E. coli* cell membranes expressing AO3-A1 showed both a depression of the initial state absorption and the formation of two distinctive photointermediates at shorter (410 nm) and longer (640 nm) wavelengths (Figure 4A) as reported previously (Clair et al., 2012; Ernst et al., 2014; Inoue et al., 2015; Takayama et al., 2018). Judging from the time region and the location of the absorption maxima (Figure 4A), they have been assigned as M and O intermediates, respectively (Clair et al., 2012; Ernst et al., 2014; Inoue et al., 2015; Takayama et al., 2018). Similarly, upon illumination with 505 nm light, *E. coli* cellular membranes expressing AO3-DMP showed both a depression of the initial state absorption and the formation of two distinctive photointermediates at shorter (390 nm) and longer (580 nm) wavelengths (Figure 4B). We tentatively assigned them as M and O intermediates, respectively. The M intermediate was formed with the decrease in the initial state absorption soon after the flash excitation and then decayed. After that, the O intermediate was formed and then decayed with the recovery of the initial state and the cyclic reaction was completed (Figures 4C,D). The previous studies showed that AR3 forms the photointermediates in the order of M (∼400 nm), N (∼540 nm) and O (∼640 nm) intermediates and then returns to the initial state in the photocycle (Clair et al., 2012; Maclaurin et al., 2013). To analyze the reaction kinetics more precisely, the data were fitted with the exponential decay function with a sum of three exponential terms, τ_1_ for the M intermediate, τ_2_ for the N intermediate and τ_3_ for the O intermediate (Figures 4C,D), and the fitting errors were also calculated. From that analysis, we found that the photocycle kinetics of AO3-DMP (τ_1_ = 1.53 ± 0.128 ms, τ_2_ = 4.97 ± 0.927 ms, τ_3_ = 883 ± 21.1 ms) were different from those of AO3-A1 (τ_1_ = 1.42 ± 0.0138 ms, τ_2_ = 13.8 ± 0.692 ms, τ_3_ = 83.8 ± 2.28 ms), especially regarding the slow decay of the O intermediate. These data are listed in Table 1 for comparison and the photocycle model is shown as a schematic drawing in Figure 4E.

In general, the photocycle of microbial rhodopsin is driven by retinal photoisomerization (all-*trans* to 13-*cis*) (Ernst et al., 2014). AO3-DMP showed the same photointermediates as AO3-A1 reported previously (Clair et al., 2012; Maclaurin et al., 2013), therefore, DMP-retinal can absorb electromagnetic radiation and induce photoisomerization as does A1-retinal. However, the photocycling rate of AO3-DMP was 20 times slower than that of AO3-A1, especially the decay of the O-intermediate (Table 1). In the case of bacteriorhodopsin which is a homologous protein of AR3, the O-intermediate exhibits the deformation of helix C and the FG loop, and disruption of the proton-release complex (Glu194/Glu204) on the extracellular side compared with the initial state (Zhang et al., 2012). Then, the deformation is relaxed and a proton is transferred from the counterion (Asp95) to the proton-release complex during the decay of the O-intermediate into the initial state (Kandori, 2004; Zhang et al., 2012; Ernst et al., 2014). As a future work, we need to investigate the structural changes of helix C, the FG loop and proton-release complex in the O-intermediate of AO3-DMP by using vibrational spectroscopic and/or X-ray crystallography. The structural information will allow us to identify key structural elements, regulating the slow decay rate of the O-intermediate of AO3-DMP. In addition, it is known that a proton is transferred from the intracellular to the extracellular side during a single photocycle in proton pump rhodopsins. Thus, the photocycling rate is tightly coupled with the proton pumping activity. We hypothesize that the decrease of the photocycling rate leads to the low photo-induced proton pump activity of AO3-DMP, as shown in Figure 2D.

As described above, we observed delayed recovery of pH changes for AO3-DMP with the turn-off of the light in the proton transport measurements (Figure 2C). Just when light was turned off, a part of AO3-DMP pigments started its photocycle. After that, the pigments transported substrate protons during the slow photocycle despite turn-off of the light. We hypothesize that the prolonged pH changes were due to the slow photocycle of AO3-DMP. On the other hand, it is possible that the photoisomerization of DMP-retinal in AO3 is different from that of A1-retinal (i.e., the photoisomerization of all-*trans* to 13-*cis* form and the following thermal isomerization from 13-*cis* to all-*trans* form during the photocycle), which could lead to the slow photocycle and prolonged pH changes in DMP-AO3. As a future work, we need to investigate isomeric forms of DMP-AO3 in the dark and during the photocycle by using vibrational spectroscopic measurements and high-performance liquid chromatography (HPLC) analysis.

### Reaction With Hydroxylamine

**FIGURE 5:**
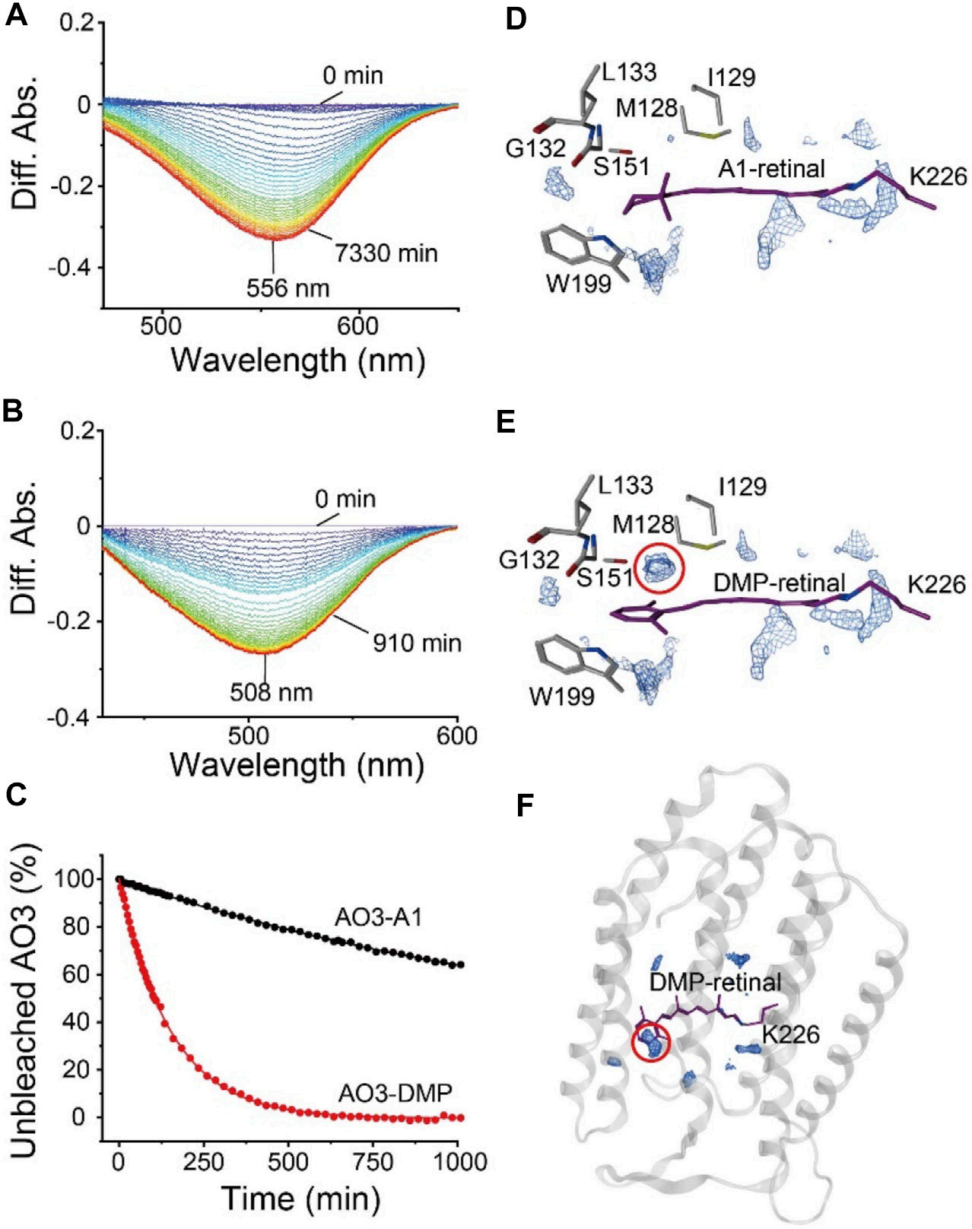
The bleaching kinetics of AO3 with A1-retinal or DMP-retinal, induced by reaction with hydroxylamine. **(A,B)** Absorption spectra were recorded at various time points after the addition of hydroxylamine (100 mM) in *E. coli* membranes at room temperature (approx. 28°C). Difference spectra at each time point taking the spectra recorded at 0 min as a reference. The peaks at 556 and 508 nm correspond to the absorption maximum of the unbleached states of AO3-A1 **(A)** and AO3-DMP **(B)**. **(C)** Bleaching kinetics in the dark. The differences in absorption at 556 nm (AO3-A1) and at 508 nm (AO3-DMP) were plotted against their respective times. The data were fitted with a single-exponential equation to estimate the rate constants. **(D,E)** Distribution pattern of water molecules (blue mesh) in the QM/MM-optimized AO3-A1 **(D)** and AO3-DMP structures **(E)** calculated using a three-dimensional reference interaction site model (3D-RISM) (Beglov and Roux, 1997; Kovalenko and Hirata, 1999; Luchko et al., 2010; Case et al., 2012; Sindhikara et al., 2012). **(F)** Overview of the QM/MM-optimized AO3-DMP structure. The red dotted circle indicates the cavity space at the aromatic ring moiety, which is absent at the corresponding *β*-ionone moiety in AO3-A1 structure.

To investigate the structure around the Schiff base of the chromophore, we measured the reactivity with the water-soluble reagent hydroxylamine as described previously (Kojima et al., 2020a). Although microbial rhodopsins are membrane proteins, hydroxylamine has been shown to be able to react with them: hydroxylamine attacks the Schiff base and thus bleaches the pigment (Subramaniam et al., 1991; Rousso et al., 1998; Sanz et al., 1999; Iwamoto et al., 2001; Sudo et al., 2002). The increase in reactivity with such reagents is mainly caused by an increase in the accessibility of hydroxylamine to the relatively hydrophilic Schiff base region *via* a narrow hydrophilic channel or cavity (see Figure 1B) (Iwamoto et al., 2001; Sudo et al., 2002). Therefore, the reactivity with hydroxylamine is a monitor of the environment and the environmental changes in the region around the Schiff base. Figure 5 shows the difference absorption spectra of AO3-A1 (Figure 5A) and AO3-DMP (Figure 5B) in *E. coli* membranes at a number of time points after the addition of excess amount of hydroxylamine (100 mM) in the dark at room temperature (approx. 28°C). As can be seen, there is a decrease in absorbance at 556 nm (AO3-A1) and 508 nm (AO3-DMP), while no spectral changes were observed in the absence of hydroxylamine. The absorbance changes at 556 nm (AO3-A1) and at 508 nm (AO3-DMP) were then plotted against time (closed circles in Figure 5C). These data were well fitted with a single-exponential equation by the first-order reactions (solid lines in Figure 5C). The bleaching rates and their fitting errors were calculated to be 3.91 × 10^–4^ ± 4.08 × 10^–6^ and 6.61 × 10^–3^ ± 4.08 × 10^–4^ min^−1^ for AO3-A1 and AO3-DMP, respectively, and are listed in Table 1 for comparison. As seen, the bleaching rate for AO3-DMP was approximately 17-fold larger than that of AO3-A1. It has been estimated that hydroxylamine attacks the protonated retinal Schiff base from the relatively hydrophilic extracellular side (Subramaniam et al., 1991; Jastrzebska et al., 2011). The QM/MM calculations show that a cavity space is present at the aromatic ring moiety in the AO3-DMP structure whereas it is absent at the corresponding *β*-ionone ring moiety in the AO3-A1 structure (Figures 5D–F). The presence of the cavity space is likely to facilitate the accessibility of hydroxylamine into the AO3-DMP moiety, substantially increasing the overall hydrophilicity.

**FIGURE 6:**
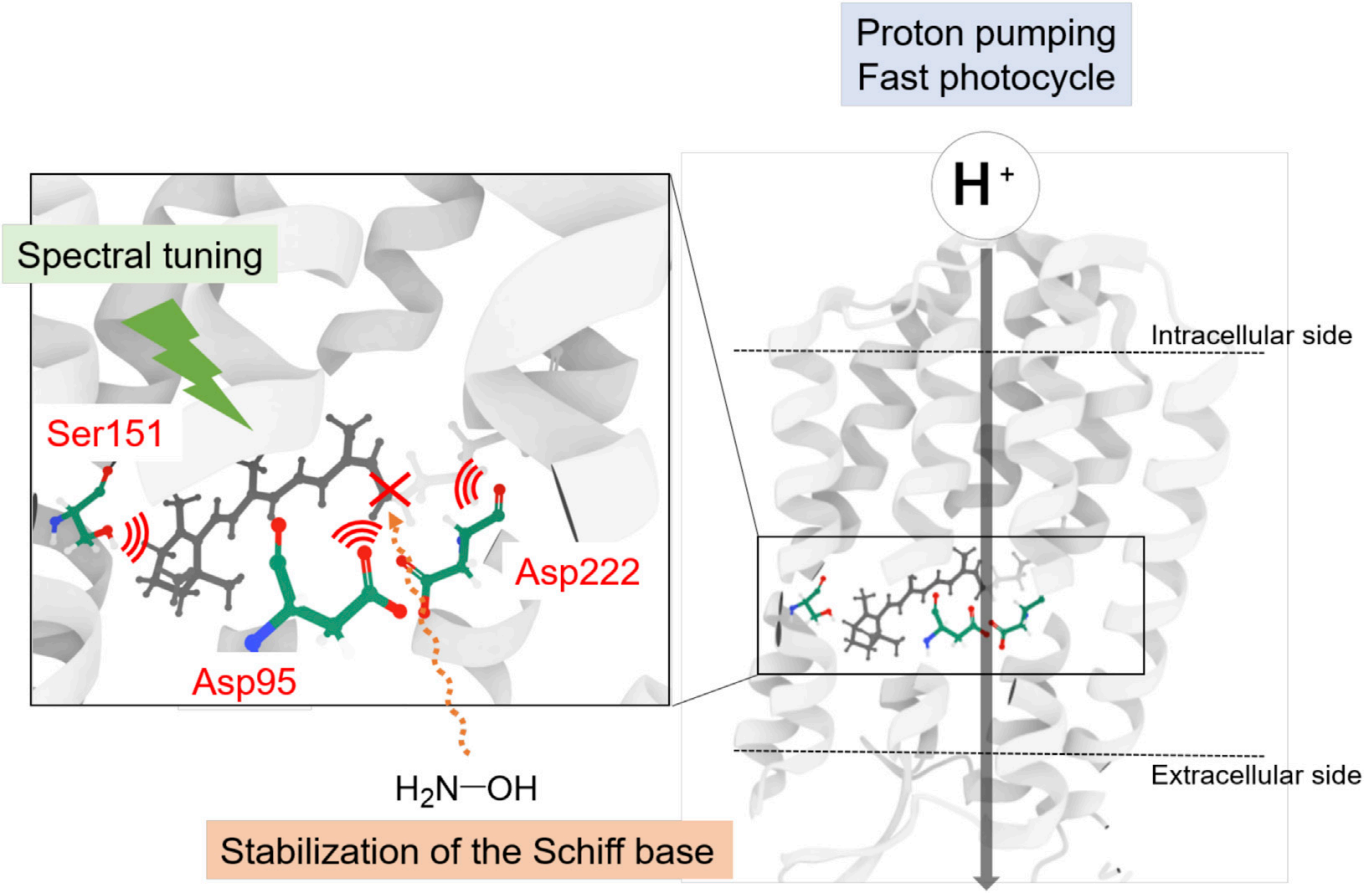
Schematics of the chromophore binding cavity of AR3. The natural protein AR3 was optimized by nature to have a tuned absorption maximum, a resistance against bleaching of the chromophore.

In this study, we synthesized the retinal analog DMP-retinal to explore the chromophore binding cavity of AR3, a light-driven outward proton pump utilized for optogenetics. The results show that DMP-retinal was successfully incorporated into the apoprotein of AR3 (AO3) and had several significant effects on the photochemical properties as follows: 1) the spectroscopic measurements revealed that the absorption maximum of AO3-DMP was dramatically blue-shifted to 508 nm. The spectral shift is due to the significant increase in the HOMO-LUMO energy gap of the chromophore with the contribution of some residues around the chromophore, 2) the time-resolved spectroscopic measurements revealed that the photocycling rate of AO3-DMP was significantly decreased, and 3) kinetical spectroscopic measurements revealed that the sensitivity of the chromophore to reaction with hydroxylamine was significant increased. The QM/MM calculations show that a cavity space is present at the aromatic ring moiety in the AO3-DMP structure whereas it is absent at the corresponding *β*-ionone ring moiety in the AO3-A1 structure. Thus, the natural protein AR3 was tuned to have a green-sensitive absorption maximum, a resistance against bleaching of the chromophore (Figure 6). To develop new optogenetics tools, many variants of rhodopsins with characteristic properties caused by introducing mutations and retinal analogs have been produced (Sineshchekov et al., 2012; Azimihashemi et al., 2014; Ganapathy et al., 2017; Herwig et al., 2017; Kaneko et al., 2017; Wiegert et al., 2017; Ganapathy et al., 2019; Mei et al., 2020). Noteworthy, AO3-DMP showed its slow photocycle (Figure 4). AR3 shows voltage-dependent fluorescence and has been used as a voltage indicator (Kralj et al., 2011; Flytzanis et al., 2014; Kojima et al., 2020a). DMP-AO3 shows the slow photocycle, which leads to the more accumulation of the intermediate states during the continuous irradiation for visualization of the fluorescence. Since the fluorescence originates from the fluorescence of the intermediate, we speculate that DMP-AO3 shows brighter fluorescence as compared with AR3. As a future work, we need to investigate the applicability of DMP-AO3 as a high fluorescence voltage indicator.

## Data Availability

The original contributions presented in the study are included in the article/Supplementary Material, further inquiries can be directed to the corresponding author.
