# Supplementary material for: Exploring the Retinal Binding Cavity of Archaerhodopsin-3 by Replacing the Retinal Chromophore With a Dimethyl Phenylated Derivative
Source: Front Mol Biosci. 2021 Dec 20;8:794948. doi: 10.3389/fmolb.2021.794948 (PMC8721008; doi:10.3389/fmolb.2021.794948)
Supplement: Supplementary file 1 [file DataSheet2.docx]

Supplementary Material

Exploring the retinal binding cavity of archaerhodopsin-3 by replacing the retinal chromophore with a dimethyl phenylated derivative

Taichi Tsuneishi^1,‡^, Masataka Takahashi^2,‡^, Masaki Tsujimura^3,‡^, Keiichi Kojima^1^, Hiroshi Ishikita^3,4^, Yasuo Takeuchi^2^ and Yuki Sudo^1,*^

^1^Laboratory of Biophysical Chemistry, Graduate School of Medicine, Dentistry and Pharmaceutical Sciences, Okayama University, Okayama 700-8530, Japan.

^2^Laboratory of Synthetic and Medicinal Chemistry, Graduate School of Medicine, Dentistry and Pharmaceutical Sciences, Okayama University, Okayama 700-8530, Japan.

^3^Department of Applied Chemistry, The University of Tokyo, Tokyo 113-8654, Japan.

^4^Research Center for Advanced Science and Technology, The University of Tokyo, Tokyo 153-8904, Japan.

*** Correspondence:**Yuki Sudo, PhD
Email: sudo@okayama-u.ac.jp

Supplementary Figures 1-6

Supplementary Table 1

Supplementary References

# Supplementary Figures

**Supplementary Figure 1.** Synthetic pathway of a dimethyl phenylated retinal derivative (DMP-retinal). (2E,4E,6E,8E)-9-(2,6-Dimethylphenyl)-3,7-dimethylnona-2,4,6,8-tetraenal (DMP-retinal) used in this study was synthesized from 2,6-dimethylbenzaldehyde as a versatile synthon. The overall yield was estimated to be approximately 17 %.


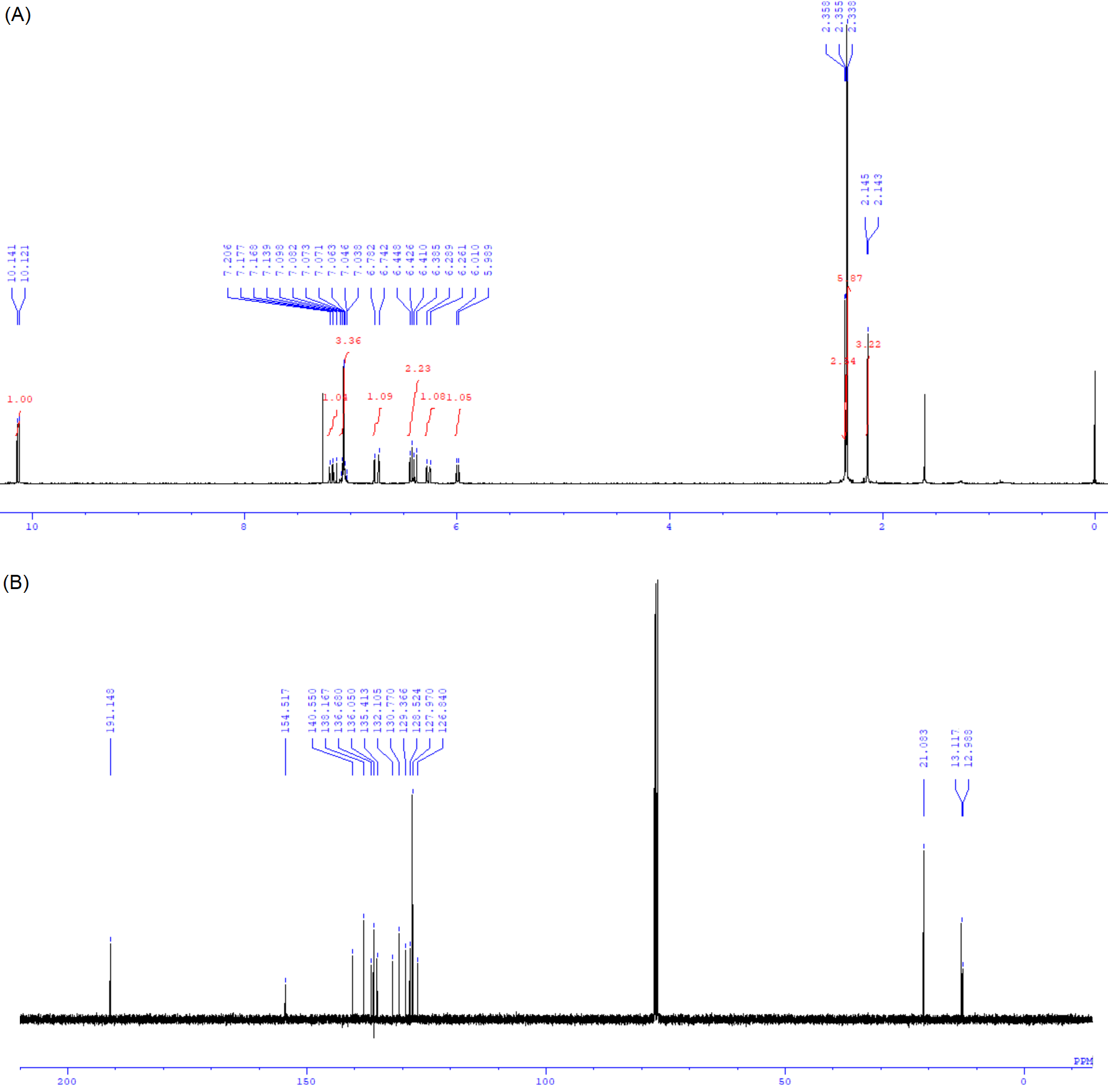


**Supplementary Figure 2.** NMR spectra of DMP-retinal. (A) ^1^H NMR, 400 MHz, in CDCl_3_. (B) ^13^C NMR, 100 MHz, in CDCl_3._ Orange crystals; mp 109.6-113.5ºC; ^1^H-NMR (CDCl_3_) δ: 2.14 (3H, d, *J* = 1.0 Hz, C(17)-*H*_3_), 2.34 (6H, s, C(18)-*H*_3_, C(19)-*H*_3_), 2.36 (3H, d, *J* = 1.2 Hz, C(16)-*H*_3_), 6.00 (1H, dq, *J* = 8.1, 1.0 Hz, C(2)-*H*), 6.28 (1H, dq, *J* = 11.4, 1.2 Hz, C(6)-*H*), 6.41 (1H, d, *J* = 16.3 Hz, C(8)-*H*), 6.43 (1H, d, *J* = 15.2 Hz, C(4)-*H*), 6.76 (1H, d, *J* = 16.3 Hz, C(9)-*H*), 7.04–7.10 (3H, m, C(12)-*H*, C(13)-*H*, C(14)-*H*), 7.17 (1H, dd, *J* = 15.2, 11.4 Hz, C(5)-*H*), 10.13 (1H, d, *J* = 8.1 Hz, C(1)-*H*O); ^13^C-NMR (100 MHz, CDCl_3_) δ: 191.15, 154.52, 140.55, 138.17, 136.68, 136.05, 135.41, 132.11, 130.77, 129.37, 128.52, 127.97, 126.84, 21.08, 13.12, 12.99.; *Anal.* Calc’d for C_19_H_22_O: C, 85.67; H, 8.32; O, 6.01. Found: C, 85.57; H, 8.29.


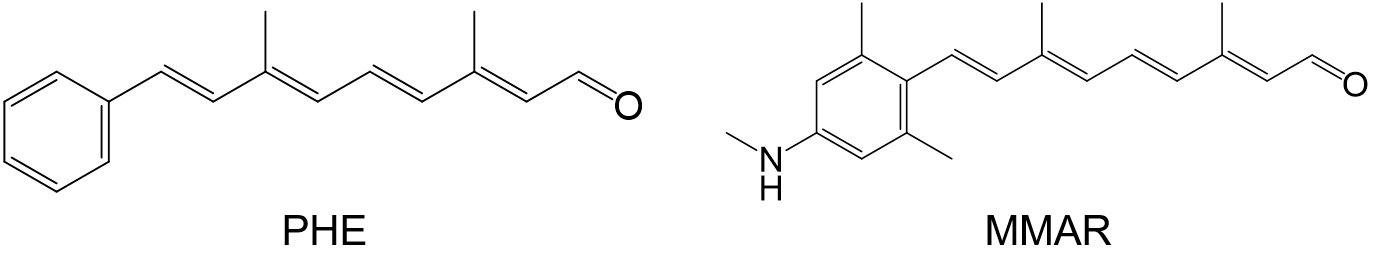


**Supplementary Figure 3.** Chemical structures of (2E,4E,6E,8E)-9-phenyl-3,7-dimethylnona-2,4,6,8-tetraenal (named PHE) and (2E,4E,6E,8E)-9-(2,6-Dimethyl-4-methylamino)phenyl-3,7-dimethylnona-2,4,6,8-tetraenal (named MMAR).

**Supplementary Figure 4.** The negative binding free-energy map of the retinal to AO3 in the QM/MM-optimized AO3-DMP, calculated using 3D-RISM. The iso-level is set to −0.25 kcal mol^−1^ Å^−3^. Red dotted circles indicate negative binding free-energy spaces near the methyl groups.


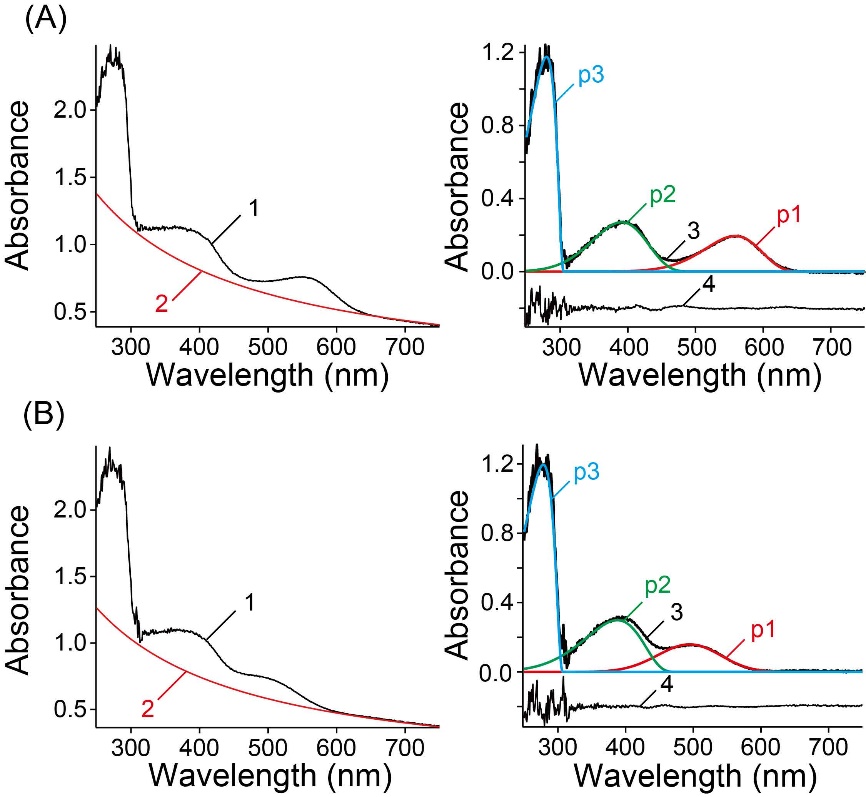


**Supplementary Figure 5.** Estimation of the amounts of photoactive proteins in *E. coli* cells. (A, B) Estimation of the total amounts of photoactive proteins in *E. coli* cells expressing AO3-A1 (A) and AO3-DMP (B). Curve 1 is the absorption spectrum of the suspension of cell membranes. The contribution of the background light scattering (curve 2) was subtracted from curve 1 to obtain the spectrum consisting of the absorption of photoactive proteins and contaminated proteins (curve 3). Curve 3 was fitted by three log-normal equations (p1, p2 and p3). Curve 4 is the residual spectrum between curve 3 and the fitting curve. From the absorbance of the main band (p1) at 558 and 494 nm for AO3-A1 and AO3-DMP, respectively, the total amounts of photoactive proteins were estimated as previously described ([Kojima et al., 2020](#_ENREF_1)).

**Supplementary Figure 6.** (A) S_0_ and (B) S_1_ state charges of the retinal Schiff base calculated for the A1- and DMP-retinal Schiff bases in the absence of the protein environment. The DFT method was employed ﻿with the B3LYP functional and 6-31G* basis sets using the GAUSSIAN 16 program (Frisch et al., 2016). (C) Chemical structure of retinal Schiff base. (D) Electrostatic effects of counterions on the energies of the S_0_ and S_1_ states in the AO3-A1 and AO3-DMP structures.

# Supplementary Table

**Table 1.** Calculated (calc.) and experimentally measured (expl.) absorption wavelengths for A1- and DMP-retinals (or retinal Schiff bases) in the absence of the protein environment (nm).

|  | **environment** | **chromophore** | **A1-retinal** | **DMP-retinal** |
| --- | --- | --- | --- | --- |
| expl. | in ethanol | retinal | 383.5 | 377 |
| calc. (1) ^a^ | in vacuum | retinal Schiff base | 671 | 658 |
| calc. (2) ^b^ | in vacuum | retinal Schiff base | 645 | 639 |
| calc. (3) ^b^ | in vacuum | retinal | 423 | 398 |

^a^ Calculated using the QM/MM-optimized structures. ^b^ Calculated using the geometries optimized in vacuum.

**Table 2.** Calculated energy levels of the HOMO and LUMO and the absorption energies (*E*_abs_) in AO3-A1 and AO3-DMP (eV).

|  | AO3-A1 |  |  | AO3-DMP |  |  |
| --- | --- | --- | --- | --- | --- | --- |
|  | HOMO | LUMO | *E*_abs_ ^a^ | HOMO | LUMO | *E*_abs_ ^a^ |
| wild-type | −4.751 | −2.370 | 2.220 | −4.996 | −2.493 | 2.387 |
| w/o Asp222 ^b^ | −5.763 | −3.510 | 2.046 | −5.946 | −3.619 | 2.146 |
| w/o Asp95 ^b^ | −5.521 | −3.293 | 2.013 | −5.712 | −3.401 | 2.124 |
| w/o Ser151 ^b^ | −4.920 | −2.495 | 2.280 | −5.129 | −2.593 | 2.431 |

^a^Empirically corrected using eq. 1. ^b^Calculated in the absence of the atomic partial charges of the focusing side-chain.

# Supplementary References

Kojima, K., Ueta, T., Noji, T., Saito, K., Kanehara, K., Yoshizawa, S., Ishikita, H., and Sudo, Y. (2020). Vectorial proton transport mechanism of RxR, a phylogenetically distinct and thermally stable microbial rhodopsin. *Sci. Rep.* 10**,** 282. doi: 10.1038/s41598-019-57122-2.

Frisch, M. J., Trucks, G. W., Schlegel, H. B., Scuseria, G. E., Robb, M. A., Cheeseman, J. R., et al. (2016). Gaussian 16 Rev. A.03, Wallingford, CT.
