# Supplementary material for: Exploring the Retinal Binding Cavity of Archaerhodopsin-3 by Replacing the Retinal Chromophore With a Dimethyl Phenylated Derivative
Source: Front Mol Biosci. 2021 Dec 20;8:794948. doi: 10.3389/fmolb.2021.794948 (PMC8721008; doi:10.3389/fmolb.2021.794948)
Supplement: Supplementary file 2 [file DataSheet1.docx]

TITLE 1 SUPPORTING INFORMATION FOR:

TITLE 2 EXPLORING THE RETINAL BINDING CAVITY OF ARCHAERHODOPSIN-3 BY REPLACING THE RETINAL CHROMOPHORE WITH A DIMETHYL PHENYLATED DERIVATIVE

TITLE 3 T.TSUNEISHI, M.TAKAHASHI, M.TSUJIMURA, K.KOJIMA, H.ISHIKITA, Y.TAKEUCHI, Y.SUDO

REMARK 400

REMARK 400 TABLE OF CONTENTS

REMARK 400 GEOMETRIES OF THE QMMM-OPTIMIZED AR3 STRUCTURES

REMARK 400

REMARK 400 MODEL 1:AO3 WITH ALL-TRANS RETINAL

REMARK 400 MODEL 2:AO3 WITH DMP-RETINAL

REMARK 400

REMARK 400

MODEL 1

ATOM 1 CB ARG A 92 1.646 2.944 -20.406 1.00 12.44 C

ATOM 2 CG ARG A 92 0.430 2.194 -20.994 1.00 13.66 C

ATOM 3 CD ARG A 92 -0.030 0.987 -20.209 1.00 15.36 C

ATOM 4 NE ARG A 92 0.838 -0.168 -20.421 1.00 17.74 N

ATOM 5 CZ ARG A 92 1.754 -0.585 -19.541 1.00 16.95 C

ATOM 6 NH1 ARG A 92 2.896 -1.049 -20.013 1.00 16.17 N

ATOM 7 NH2 ARG A 92 1.495 -0.599 -18.193 1.00 19.02 N1+

ATOM 8 1HB ARG A 92 1.360 3.389 -19.452 1.00 12.22 H

ATOM 9 2HB ARG A 92 2.471 2.250 -20.204 1.00 12.22 H

ATOM 10 1HG ARG A 92 0.612 1.857 -22.020 1.00 13.80 H

ATOM 11 2HG ARG A 92 -0.455 2.841 -21.025 1.00 13.68 H

ATOM 12 1HD ARG A 92 -1.016 0.746 -20.608 1.00 15.28 H

ATOM 13 2HD ARG A 92 -0.111 1.224 -19.146 1.00 15.29 H

ATOM 14 HE ARG A 92 1.156 -0.273 -21.395 1.00 17.18 H

ATOM 15 1HH1 ARG A 92 3.093 -0.850 -21.010 1.00 16.33 H

ATOM 16 2HH1 ARG A 92 3.610 -1.490 -19.442 1.00 16.38 H

ATOM 17 1HH2 ARG A 92 0.635 -0.128 -17.930 1.00 18.62 H

ATOM 18 2HH2 ARG A 92 2.306 -0.216 -17.682 1.00 18.54 H

ATOM 19 HCAP ARG A 92 2.071 3.731 -21.057 1.00 12.44 H

ATOM 20 CB TYR A 93 -1.870 6.563 -21.624 1.00 10.19 C

ATOM 21 CG TYR A 93 -2.523 5.469 -20.800 1.00 10.31 C

ATOM 22 CD1 TYR A 93 -3.617 4.748 -21.303 1.00 10.81 C

ATOM 23 CE1 TYR A 93 -4.144 3.661 -20.610 1.00 11.21 C

ATOM 24 CZ TYR A 93 -3.577 3.278 -19.392 1.00 11.65 C

ATOM 25 OH TYR A 93 -4.106 2.187 -18.758 1.00 13.61 O

ATOM 26 CD2 TYR A 93 -2.001 5.091 -19.555 1.00 10.81 C

ATOM 27 CE2 TYR A 93 -2.514 4.004 -18.850 1.00 11.67 C

ATOM 28 1HB TYR A 93 -2.618 7.086 -22.229 1.00 10.06 H

ATOM 29 2HB TYR A 93 -1.374 7.304 -20.989 1.00 10.09 H

ATOM 30 HD1 TYR A 93 -4.063 5.037 -22.252 1.00 10.70 H

ATOM 31 HE1 TYR A 93 -4.991 3.106 -20.998 1.00 11.21 H

ATOM 32 HH TYR A 93 -3.532 1.927 -17.997 1.00 13.56 H

ATOM 33 HD2 TYR A 93 -1.169 5.642 -19.123 1.00 10.79 H

ATOM 34 HE2 TYR A 93 -2.084 3.725 -17.894 1.00 11.29 H

ATOM 35 HCAP TYR A 93 -1.117 6.114 -22.293 1.00 10.19 H

ATOM 36 CB ASP A 95 4.001 5.018 -25.488 1.00 12.04 C

ATOM 37 CG ASP A 95 4.425 3.738 -26.266 1.00 15.21 C

ATOM 38 OD1 ASP A 95 4.195 3.535 -27.480 1.00 14.84 O

ATOM 39 OD2 ASP A 95 5.068 2.925 -25.511 1.00 13.45 O1-

ATOM 40 1HB ASP A 95 3.498 4.670 -24.584 1.00 12.18 H

ATOM 41 2HB ASP A 95 4.920 5.472 -25.145 1.00 11.99 H

ATOM 42 HCAP ASP A 95 3.422 5.861 -25.900 1.00 12.04 H

ATOM 43 CB TRP A 96 -0.904 3.610 -26.447 1.00 8.38 C

ATOM 44 CG TRP A 96 -0.332 2.384 -25.790 1.00 9.09 C

ATOM 45 CD1 TRP A 96 0.826 1.681 -26.040 1.00 10.02 C

ATOM 46 NE1 TRP A 96 0.890 0.555 -25.237 1.00 10.92 N

ATOM 47 CE2 TRP A 96 -0.232 0.508 -24.449 1.00 9.86 C

ATOM 48 CD2 TRP A 96 -1.017 1.656 -24.759 1.00 9.07 C

ATOM 49 CE3 TRP A 96 -2.202 1.891 -24.040 1.00 10.17 C

ATOM 50 CZ3 TRP A 96 -2.594 0.977 -23.071 1.00 10.92 C

ATOM 51 CZ2 TRP A 96 -0.636 -0.425 -23.486 1.00 9.83 C

ATOM 52 CH2 TRP A 96 -1.832 -0.185 -22.817 1.00 11.14 C

ATOM 53 1HB TRP A 96 -1.750 3.317 -27.077 1.00 8.44 H

ATOM 54 2HB TRP A 96 -1.304 4.294 -25.688 1.00 8.49 H

ATOM 55 HD1 TRP A 96 1.636 1.904 -26.722 1.00 9.93 H

ATOM 56 HE1 TRP A 96 1.598 -0.177 -25.294 1.00 10.38 H

ATOM 57 HE3 TRP A 96 -2.794 2.782 -24.225 1.00 10.04 H

ATOM 58 HZ3 TRP A 96 -3.494 1.159 -22.497 1.00 10.40 H

ATOM 59 HZ2 TRP A 96 -0.034 -1.300 -23.271 1.00 9.88 H

ATOM 60 HH2 TRP A 96 -2.179 -0.903 -22.079 1.00 10.54 H

ATOM 61 HCAP TRP A 96 -0.205 4.190 -27.064 1.00 8.38 H

ATOM 62 CB THR A 99 3.190 4.022 -30.948 1.00 10.49 C

ATOM 63 OG1 THR A 99 2.519 3.443 -29.840 1.00 11.23 O

ATOM 64 CG2 THR A 99 4.455 4.812 -30.578 1.00 12.29 C

ATOM 65 HB THR A 99 3.493 3.200 -31.611 1.00 10.41 H

ATOM 66 HG1 THR A 99 2.987 3.709 -29.019 1.00 11.16 H

ATOM 67 1HG2 THR A 99 4.928 5.251 -31.461 1.00 11.97 H

ATOM 68 2HG2 THR A 99 5.183 4.159 -30.094 1.00 12.12 H

ATOM 69 3HG2 THR A 99 4.242 5.623 -29.876 1.00 11.90 H

ATOM 70 HCAP THR A 99 2.529 4.655 -31.570 1.00 10.49 H

ATOM 71 CB THR A 100 -2.241 2.912 -31.553 1.00 9.31 C

ATOM 72 OG1 THR A 100 -2.881 3.858 -30.733 1.00 10.41 O

ATOM 73 CG2 THR A 100 -1.648 1.788 -30.697 1.00 10.25 C

ATOM 74 HB THR A 100 -3.036 2.481 -32.166 1.00 9.41 H

ATOM 75 HG1 THR A 100 -2.217 4.330 -30.186 1.00 10.37 H

ATOM 76 1HG2 THR A 100 -1.239 0.977 -31.308 1.00 10.05 H

ATOM 77 2HG2 THR A 100 -2.439 1.371 -30.070 1.00 10.02 H

ATOM 78 3HG2 THR A 100 -0.851 2.155 -30.040 1.00 10.16 H

ATOM 79 HCAP THR A 100 -1.474 3.287 -32.264 1.00 9.31 H

ATOM 80 CB LEU A 103 0.698 0.936 -35.861 1.00 9.38 C

ATOM 81 CG LEU A 103 1.981 0.626 -35.055 1.00 10.24 C

ATOM 82 CD1 LEU A 103 1.610 0.091 -33.666 1.00 10.75 C

ATOM 83 CD2 LEU A 103 2.871 -0.375 -35.796 1.00 10.59 C

ATOM 84 1HB LEU A 103 0.287 -0.007 -36.249 1.00 9.28 H

ATOM 85 2HB LEU A 103 -0.064 1.371 -35.203 1.00 9.65 H

ATOM 86 HG LEU A 103 2.550 1.550 -34.900 1.00 10.22 H

ATOM 87 1HD1 LEU A 103 1.077 0.847 -33.082 1.00 10.68 H

ATOM 88 2HD1 LEU A 103 2.507 -0.203 -33.109 1.00 10.72 H

ATOM 89 3HD1 LEU A 103 0.966 -0.795 -33.739 1.00 10.65 H

ATOM 90 1HD2 LEU A 103 2.287 -1.261 -36.073 1.00 10.58 H

ATOM 91 2HD2 LEU A 103 3.704 -0.726 -35.180 1.00 10.53 H

ATOM 92 3HD2 LEU A 103 3.294 0.045 -36.715 1.00 10.50 H

ATOM 93 HCAP LEU A 103 0.828 1.646 -36.694 1.00 9.38 H

ATOM 94 CB MET A 128 -9.367 3.276 -28.610 1.00 9.00 C

ATOM 95 CG MET A 128 -8.894 1.904 -28.123 1.00 9.49 C

ATOM 96 SD MET A 128 -8.102 1.046 -29.540 1.00 10.90 S

ATOM 97 CE MET A 128 -6.428 1.762 -29.474 1.00 10.59 C

ATOM 98 1HB MET A 128 -8.554 3.677 -29.193 1.00 9.19 H

ATOM 99 2HB MET A 128 -10.186 3.142 -29.318 1.00 8.94 H

ATOM 100 1HG MET A 128 -9.709 1.270 -27.783 1.00 9.76 H

ATOM 101 2HG MET A 128 -8.161 1.937 -27.312 1.00 9.62 H

ATOM 102 1HE MET A 128 -5.867 1.339 -30.309 1.00 10.70 H

ATOM 103 2HE MET A 128 -6.443 2.849 -29.579 1.00 10.57 H

ATOM 104 3HE MET A 128 -5.932 1.485 -28.543 1.00 10.64 H

ATOM 105 HCAP MET A 128 -9.676 4.057 -27.898 1.00 9.00 H

ATOM 106 CB ILE A 129 -5.308 6.001 -26.644 1.00 9.62 C

ATOM 107 CG2 ILE A 129 -4.168 6.392 -25.675 1.00 10.13 C

ATOM 108 CG1 ILE A 129 -4.773 5.010 -27.710 1.00 9.93 C

ATOM 109 CD1 ILE A 129 -4.415 3.603 -27.224 1.00 11.01 C

ATOM 110 HB ILE A 129 -5.623 6.906 -27.180 1.00 9.47 H

ATOM 111 1HG2 ILE A 129 -4.353 7.327 -25.134 1.00 9.89 H

ATOM 112 2HG2 ILE A 129 -3.248 6.535 -26.239 1.00 10.16 H

ATOM 113 3HG2 ILE A 129 -3.963 5.615 -24.929 1.00 10.11 H

ATOM 114 1HG1 ILE A 129 -3.876 5.464 -28.145 1.00 9.96 H

ATOM 115 2HG1 ILE A 129 -5.462 4.918 -28.550 1.00 9.88 H

ATOM 116 HD1 ILE A 129 -3.763 3.625 -26.347 1.00 10.80 H

ATOM 117 HD2 ILE A 129 -3.897 3.061 -28.022 1.00 10.78 H

ATOM 118 HD3 ILE A 129 -5.302 3.018 -26.957 1.00 10.79 H

ATOM 119 HCAP ILE A 129 -6.213 5.613 -26.128 1.00 9.62 H

ATOM 120 CB TRP A 148 -12.540 -1.195 -19.605 1.00 11.57 C

ATOM 121 CG TRP A 148 -12.177 -2.510 -20.266 1.00 11.39 C

ATOM 122 CD1 TRP A 148 -10.938 -2.869 -20.734 1.00 12.11 C

ATOM 123 NE1 TRP A 148 -10.994 -4.089 -21.381 1.00 13.19 N

ATOM 124 CE2 TRP A 148 -12.276 -4.586 -21.283 1.00 13.62 C

ATOM 125 CD2 TRP A 148 -13.054 -3.621 -20.587 1.00 11.93 C

ATOM 126 CE3 TRP A 148 -14.409 -3.907 -20.333 1.00 15.15 C

ATOM 127 CZ3 TRP A 148 -14.941 -5.113 -20.775 1.00 17.56 C

ATOM 128 CZ2 TRP A 148 -12.804 -5.801 -21.730 1.00 16.01 C

ATOM 129 CH2 TRP A 148 -14.147 -6.052 -21.468 1.00 17.33 C

ATOM 130 1HB TRP A 148 -13.377 -1.351 -18.924 1.00 11.59 H

ATOM 131 2HB TRP A 148 -11.707 -0.829 -19.000 1.00 11.56 H

ATOM 132 HD1 TRP A 148 -10.003 -2.338 -20.658 1.00 12.33 H

ATOM 133 HE1 TRP A 148 -10.182 -4.676 -21.550 1.00 12.74 H

ATOM 134 HE3 TRP A 148 -15.033 -3.197 -19.798 1.00 14.70 H

ATOM 135 HZ3 TRP A 148 -15.985 -5.341 -20.582 1.00 16.70 H

ATOM 136 HZ2 TRP A 148 -12.183 -6.524 -22.251 1.00 15.56 H

ATOM 137 HH2 TRP A 148 -14.591 -6.987 -21.798 1.00 16.25 H

ATOM 138 HCAP TRP A 148 -12.811 -0.353 -20.273 1.00 11.57 H

ATOM 139 CB SER A 151 -11.559 1.947 -24.168 1.00 9.86 C

ATOM 140 OG SER A 151 -10.242 1.829 -24.681 1.00 10.47 O

ATOM 141 1HB SER A 151 -11.717 1.083 -23.519 1.00 9.93 H

ATOM 142 2HB SER A 151 -11.684 2.835 -23.546 1.00 10.01 H

ATOM 143 HG1 SER A 151 -9.885 2.716 -24.875 1.00 10.52 H

ATOM 144 HCAP SER A 151 -12.361 1.941 -24.929 1.00 9.86 H

ATOM 145 CB THR A 152 -13.397 -2.896 -25.362 1.00 10.97 C

ATOM 146 OG1 THR A 152 -12.395 -3.002 -24.346 1.00 11.95 O

ATOM 147 CG2 THR A 152 -13.449 -4.278 -26.014 1.00 13.27 C

ATOM 148 HB THR A 152 -14.377 -2.676 -24.918 1.00 11.13 H

ATOM 149 HG1 THR A 152 -12.562 -2.305 -23.684 1.00 11.95 H

ATOM 150 1HG2 THR A 152 -14.263 -4.380 -26.739 1.00 12.88 H

ATOM 151 2HG2 THR A 152 -13.603 -5.011 -25.217 1.00 12.81 H

ATOM 152 3HG2 THR A 152 -12.504 -4.514 -26.514 1.00 12.97 H

ATOM 153 HCAP THR A 152 -13.146 -2.056 -26.043 1.00 10.97 H

ATOM 154 CB MET A 155 -10.328 -1.621 -29.630 1.00 10.21 C

ATOM 155 CG MET A 155 -9.119 -2.408 -30.159 1.00 11.14 C

ATOM 156 SD MET A 155 -8.649 -3.801 -29.082 1.00 11.57 S

ATOM 157 CE MET A 155 -10.145 -4.840 -29.267 1.00 12.74 C

ATOM 158 1HB MET A 155 -11.069 -2.294 -29.196 1.00 10.18 H

ATOM 159 2HB MET A 155 -10.003 -0.948 -28.834 1.00 10.42 H

ATOM 160 1HG MET A 155 -8.239 -1.763 -30.208 1.00 11.23 H

ATOM 161 2HG MET A 155 -9.263 -2.825 -31.158 1.00 11.12 H

ATOM 162 1HE MET A 155 -10.023 -5.718 -28.632 1.00 12.51 H

ATOM 163 2HE MET A 155 -11.046 -4.312 -28.946 1.00 12.56 H

ATOM 164 3HE MET A 155 -10.261 -5.182 -30.297 1.00 12.56 H

ATOM 165 HCAP MET A 155 -10.835 -1.013 -30.393 1.00 10.21 H

ATOM 166 CB TRP A 192 -4.823 -4.459 -31.409 1.00 9.58 C

ATOM 167 CG TRP A 192 -3.722 -3.676 -32.085 1.00 10.20 C

ATOM 168 CD1 TRP A 192 -2.432 -4.033 -32.412 1.00 10.86 C

ATOM 169 NE1 TRP A 192 -1.786 -2.998 -33.061 1.00 11.18 N

ATOM 170 CE2 TRP A 192 -2.649 -1.928 -33.162 1.00 10.92 C

ATOM 171 CD2 TRP A 192 -3.879 -2.326 -32.563 1.00 10.74 C

ATOM 172 CE3 TRP A 192 -4.968 -1.435 -32.565 1.00 10.97 C

ATOM 173 CZ3 TRP A 192 -4.801 -0.174 -33.119 1.00 11.88 C

ATOM 174 CZ2 TRP A 192 -2.476 -0.652 -33.713 1.00 11.97 C

ATOM 175 CH2 TRP A 192 -3.562 0.215 -33.671 1.00 11.71 C

ATOM 176 1HB TRP A 192 -5.481 -3.749 -30.895 1.00 9.51 H

ATOM 177 2HB TRP A 192 -5.459 -4.998 -32.118 1.00 9.67 H

ATOM 178 HD1 TRP A 192 -1.897 -4.955 -32.243 1.00 10.59 H

ATOM 179 HE1 TRP A 192 -0.846 -3.061 -33.449 1.00 10.96 H

ATOM 180 HE3 TRP A 192 -5.926 -1.730 -32.145 1.00 10.96 H

ATOM 181 HZ3 TRP A 192 -5.628 0.526 -33.127 1.00 11.78 H

ATOM 182 HZ2 TRP A 192 -1.535 -0.348 -34.158 1.00 11.76 H

ATOM 183 HH2 TRP A 192 -3.453 1.220 -34.057 1.00 11.52 H

ATOM 184 HCAP TRP A 192 -4.452 -5.170 -30.656 1.00 9.58 H

ATOM 185 CB TYR A 195 -4.163 -3.968 -25.509 1.00 9.35 C

ATOM 186 CG TYR A 195 -2.744 -3.882 -26.025 1.00 8.83 C

ATOM 187 CD1 TYR A 195 -2.359 -4.591 -27.165 1.00 9.54 C

ATOM 188 CE1 TYR A 195 -1.092 -4.491 -27.718 1.00 9.39 C

ATOM 189 CZ TYR A 195 -0.142 -3.667 -27.105 1.00 8.71 C

ATOM 190 OH TYR A 195 1.066 -3.541 -27.700 1.00 9.91 O

ATOM 191 CD2 TYR A 195 -1.779 -3.073 -25.421 1.00 9.16 C

ATOM 192 CE2 TYR A 195 -0.485 -2.984 -25.929 1.00 9.21 C

ATOM 193 1HB TYR A 195 -4.337 -3.148 -24.803 1.00 9.16 H

ATOM 194 2HB TYR A 195 -4.862 -3.823 -26.333 1.00 9.38 H

ATOM 195 HD1 TYR A 195 -3.086 -5.239 -27.622 1.00 9.41 H

ATOM 196 HE1 TYR A 195 -0.823 -5.045 -28.611 1.00 9.25 H

ATOM 197 HH TYR A 195 1.652 -2.974 -27.132 1.00 9.89 H

ATOM 198 HD2 TYR A 195 -2.038 -2.470 -24.556 1.00 9.04 H

ATOM 199 HE2 TYR A 195 0.255 -2.355 -25.453 1.00 9.12 H

ATOM 200 HCAP TYR A 195 -4.405 -4.922 -25.009 1.00 9.35 H

ATOM 201 CB TRP A 199 -6.578 -3.416 -19.780 1.00 10.80 C

ATOM 202 CG TRP A 199 -6.658 -2.037 -19.190 1.00 11.22 C

ATOM 203 CD1 TRP A 199 -5.698 -1.068 -19.355 1.00 10.88 C

ATOM 204 NE1 TRP A 199 -6.117 0.134 -18.828 1.00 11.68 N

ATOM 205 CE2 TRP A 199 -7.376 -0.037 -18.300 1.00 11.95 C

ATOM 206 CD2 TRP A 199 -7.745 -1.399 -18.490 1.00 10.91 C

ATOM 207 CE3 TRP A 199 -8.979 -1.837 -17.972 1.00 11.76 C

ATOM 208 CZ3 TRP A 199 -9.791 -0.941 -17.287 1.00 12.13 C

ATOM 209 CZ2 TRP A 199 -8.214 0.878 -17.656 1.00 13.05 C

ATOM 210 CH2 TRP A 199 -9.418 0.412 -17.143 1.00 13.29 C

ATOM 211 1HB TRP A 199 -7.541 -3.694 -20.225 1.00 10.79 H

ATOM 212 2HB TRP A 199 -5.864 -3.384 -20.610 1.00 10.88 H

ATOM 213 HD1 TRP A 199 -4.737 -1.138 -19.843 1.00 11.18 H

ATOM 214 HE1 TRP A 199 -5.573 0.993 -18.854 1.00 11.36 H

ATOM 215 HE3 TRP A 199 -9.282 -2.871 -18.083 1.00 11.63 H

ATOM 216 HZ3 TRP A 199 -10.726 -1.280 -16.853 1.00 11.98 H

ATOM 217 HZ2 TRP A 199 -7.921 1.914 -17.586 1.00 12.54 H

ATOM 218 HH2 TRP A 199 -10.086 1.092 -16.623 1.00 12.83 H

ATOM 219 HCAP TRP A 199 -6.244 -4.252 -19.143 1.00 10.80 H

ATOM 220 CB ASP A 222 4.778 -2.697 -26.177 1.00 9.27 C

ATOM 221 CG ASP A 222 3.597 -1.994 -25.500 1.00 11.03 C

ATOM 222 OD1 ASP A 222 2.443 -1.956 -26.005 1.00 11.31 O

ATOM 223 OD2 ASP A 222 3.926 -1.336 -24.467 1.00 13.10 O1-

ATOM 224 1HB ASP A 222 5.335 -1.878 -26.645 1.00 9.46 H

ATOM 225 2HB ASP A 222 5.423 -3.144 -25.418 1.00 9.40 H

ATOM 226 HCAP ASP A 222 4.528 -3.457 -26.924 1.00 9.27 H

ATOM 227 CB ALA A 225 2.151 -4.423 -31.194 1.00 13.00 C

ATOM 228 1HB ALA A 225 1.422 -3.850 -31.770 1.00 12.74 H

ATOM 229 2HB ALA A 225 1.635 -5.232 -30.675 1.00 12.84 H

ATOM 230 3HB ALA A 225 2.593 -3.780 -30.429 1.00 12.85 H

ATOM 231 HCAP ALA A 225 2.930 -4.846 -31.844 1.00 13.00 H

ATOM 232 CB LYS A 226 6.488 -1.560 -31.606 1.00 11.71 C

ATOM 233 CG LYS A 226 5.302 -0.834 -30.959 1.00 11.29 C

ATOM 234 CD LYS A 226 5.704 0.274 -29.993 1.00 12.10 C

ATOM 235 CE LYS A 226 4.499 1.140 -29.643 1.00 13.89 C

ATOM 236 NZ LYS A 226 3.479 0.400 -28.909 1.00 15.97 N1+

ATOM 237 1HB LYS A 226 7.225 -0.836 -31.978 1.00 11.53 H

ATOM 238 2HB LYS A 226 7.020 -2.169 -30.870 1.00 11.56 H

ATOM 239 1HG LYS A 226 4.654 -1.544 -30.440 1.00 11.51 H

ATOM 240 2HG LYS A 226 4.665 -0.394 -31.737 1.00 11.33 H

ATOM 241 1HD LYS A 226 6.465 0.921 -30.444 1.00 12.02 H

ATOM 242 2HD LYS A 226 6.136 -0.133 -29.075 1.00 12.25 H

ATOM 243 1HE LYS A 226 4.040 1.532 -30.551 1.00 13.48 H

ATOM 244 2HE LYS A 226 4.758 1.988 -29.008 1.00 13.77 H

ATOM 245 1HZ LYS A 226 3.676 0.357 -27.886 1.00 15.85 H

ATOM 246 HCAP LYS A 226 6.134 -2.182 -32.443 1.00 11.71 H

TER 247 LYS A 226

HETATM 248 C1 RET r 300 -6.648 0.181 -23.538 1.00 9.37 C

HETATM 249 C2 RET r 300 -7.668 0.510 -22.428 1.00 9.74 C

HETATM 250 C3 RET r 300 -8.591 -0.644 -22.093 1.00 9.20 C

HETATM 251 C4 RET r 300 -9.439 -0.903 -23.328 1.00 9.43 C

HETATM 252 C5 RET r 300 -8.661 -0.904 -24.622 1.00 9.51 C

HETATM 253 C6 RET r 300 -7.369 -0.474 -24.733 1.00 9.12 C

HETATM 254 C7 RET r 300 -6.687 -0.682 -26.000 1.00 9.30 C

HETATM 255 C8 RET r 300 -5.362 -0.535 -26.275 1.00 9.01 C

HETATM 256 C9 RET r 300 -4.682 -0.938 -27.479 1.00 9.11 C

HETATM 257 C10 RET r 300 -3.324 -0.734 -27.492 1.00 8.74 C

HETATM 258 C11 RET r 300 -2.371 -1.062 -28.492 1.00 9.24 C

HETATM 259 C12 RET r 300 -1.043 -0.816 -28.249 1.00 9.25 C

HETATM 260 C13 RET r 300 0.053 -0.929 -29.150 1.00 9.08 C

HETATM 261 C14 RET r 300 1.284 -0.506 -28.670 1.00 9.47 C

HETATM 262 C15 RET r 300 2.373 -0.072 -29.439 1.00 10.61 C

HETATM 263 C16 RET r 300 -6.052 1.552 -23.940 1.00 9.20 C

HETATM 264 C17 RET r 300 -5.572 -0.766 -22.961 1.00 9.68 C

HETATM 265 C18 RET r 300 -9.477 -1.376 -25.795 1.00 9.05 C

HETATM 266 C19 RET r 300 -5.427 -1.615 -28.596 1.00 9.19 C

HETATM 267 C20 RET r 300 -0.133 -1.510 -30.520 1.00 9.42 C

HETATM 268 H21 RET r 300 -7.134 0.875 -21.542 1.00 9.59 H

HETATM 269 H22 RET r 300 -8.311 1.310 -22.809 1.00 9.74 H

HETATM 270 H31 RET r 300 -9.219 -0.389 -21.233 1.00 9.30 H

HETATM 271 H32 RET r 300 -8.018 -1.533 -21.807 1.00 9.24 H

HETATM 272 H41 RET r 300 -10.195 -0.116 -23.412 1.00 9.51 H

HETATM 273 H42 RET r 300 -10.014 -1.829 -23.231 1.00 9.33 H

HETATM 274 H71 RET r 300 -7.301 -1.077 -26.800 1.00 9.25 H

HETATM 275 H81 RET r 300 -4.702 -0.133 -25.518 1.00 9.05 H

HETATM 276 H101 RET r 300 -2.898 -0.272 -26.604 1.00 8.82 H

HETATM 277 H111 RET r 300 -2.691 -1.496 -29.434 1.00 9.21 H

HETATM 278 H121 RET r 300 -0.799 -0.400 -27.277 1.00 9.21 H

HETATM 279 H141 RET r 300 1.368 -0.344 -27.605 1.00 9.51 H

HETATM 280 H15 RET r 300 2.299 -0.008 -30.521 1.00 10.12 H

HETATM 281 H161 RET r 300 -6.832 2.168 -24.404 1.00 9.18 H

HETATM 282 H162 RET r 300 -5.213 1.498 -24.631 1.00 9.26 H

HETATM 283 H163 RET r 300 -5.699 2.077 -23.044 1.00 9.22 H

HETATM 284 H171 RET r 300 -6.015 -1.725 -22.676 1.00 9.62 H

HETATM 285 H172 RET r 300 -5.124 -0.328 -22.064 1.00 9.63 H

HETATM 286 H173 RET r 300 -4.767 -0.975 -23.667 1.00 9.66 H

HETATM 287 H181 RET r 300 -8.970 -2.075 -26.463 1.00 9.08 H

HETATM 288 H182 RET r 300 -9.797 -0.519 -26.396 1.00 9.16 H

HETATM 289 H183 RET r 300 -10.367 -1.872 -25.413 1.00 9.16 H

HETATM 290 H191 RET r 300 -5.791 -2.597 -28.274 1.00 9.14 H

HETATM 291 H192 RET r 300 -4.798 -1.762 -29.475 1.00 9.24 H

HETATM 292 H193 RET r 300 -6.303 -1.031 -28.894 1.00 9.19 H

HETATM 293 H201 RET r 300 0.685 -1.261 -31.199 1.00 9.33 H

HETATM 294 H202 RET r 300 -1.074 -1.207 -30.978 1.00 9.34 H

HETATM 295 H203 RET r 300 -0.152 -2.601 -30.417 1.00 9.44 H

HETATM 296 O HOH o 401 3.303 1.956 -23.780 1.00 15.83 O

HETATM 297 H1 HOH o 401 2.508 2.397 -24.120 1.00 0.00 H

HETATM 298 H2 HOH o 401 4.060 2.329 -24.306 1.00 0.00 H

HETATM 299 O HOH o 402 4.735 0.499 -26.509 1.00 23.77 O

HETATM 300 H1 HOH o 402 4.435 0.130 -25.657 1.00 0.00 H

HETATM 301 H2 HOH o 402 4.964 1.448 -26.285 1.00 0.00 H

HETATM 302 O HOH o 406 2.555 -0.283 -22.561 1.00 15.78 O

HETATM 303 H1 HOH o 406 2.915 -0.843 -23.304 1.00 0.00 H

HETATM 304 H2 HOH o 406 2.869 0.611 -22.857 1.00 0.00 H

ENDMDL

MODEL 2

ATOM 1 CB ARG A 92 1.659 2.944 -20.400 1.00 12.44 C

ATOM 2 CG ARG A 92 0.425 2.209 -20.963 1.00 13.66 C

ATOM 3 CD ARG A 92 -0.021 1.000 -20.177 1.00 15.36 C

ATOM 4 NE ARG A 92 0.839 -0.156 -20.417 1.00 17.74 N

ATOM 5 CZ ARG A 92 1.766 -0.590 -19.555 1.00 16.95 C

ATOM 6 NH1 ARG A 92 2.902 -1.046 -20.047 1.00 16.17 N

ATOM 7 NH2 ARG A 92 1.523 -0.622 -18.204 1.00 19.02 N1+

ATOM 8 1HB ARG A 92 1.401 3.383 -19.436 1.00 12.22 H

ATOM 9 2HB ARG A 92 2.482 2.241 -20.223 1.00 12.22 H

ATOM 10 1HG ARG A 92 0.578 1.878 -21.996 1.00 13.80 H

ATOM 11 2HG ARG A 92 -0.456 2.863 -20.967 1.00 13.68 H

ATOM 12 1HD ARG A 92 -1.015 0.771 -20.560 1.00 15.28 H

ATOM 13 2HD ARG A 92 -0.081 1.229 -19.111 1.00 15.29 H

ATOM 14 HE ARG A 92 1.142 -0.254 -21.396 1.00 17.18 H

ATOM 15 1HH1 ARG A 92 3.079 -0.843 -21.047 1.00 16.33 H

ATOM 16 2HH1 ARG A 92 3.617 -1.513 -19.498 1.00 16.38 H

ATOM 17 1HH2 ARG A 92 0.667 -0.152 -17.927 1.00 18.62 H

ATOM 18 2HH2 ARG A 92 2.339 -0.242 -17.697 1.00 18.54 H

ATOM 19 HCAP ARG A 92 2.075 3.731 -21.055 1.00 12.44 H

ATOM 20 CB TYR A 93 -1.873 6.564 -21.626 1.00 10.19 C

ATOM 21 CG TYR A 93 -2.527 5.472 -20.800 1.00 10.31 C

ATOM 22 CD1 TYR A 93 -3.618 4.746 -21.304 1.00 10.81 C

ATOM 23 CE1 TYR A 93 -4.143 3.659 -20.610 1.00 11.21 C

ATOM 24 CZ TYR A 93 -3.582 3.286 -19.387 1.00 11.65 C

ATOM 25 OH TYR A 93 -4.102 2.193 -18.750 1.00 13.61 O

ATOM 26 CD2 TYR A 93 -2.011 5.104 -19.550 1.00 10.81 C

ATOM 27 CE2 TYR A 93 -2.526 4.020 -18.842 1.00 11.67 C

ATOM 28 1HB TYR A 93 -2.621 7.086 -22.234 1.00 10.06 H

ATOM 29 2HB TYR A 93 -1.379 7.308 -20.992 1.00 10.09 H

ATOM 30 HD1 TYR A 93 -4.060 5.030 -22.256 1.00 10.70 H

ATOM 31 HE1 TYR A 93 -4.984 3.096 -21.003 1.00 11.21 H

ATOM 32 HH TYR A 93 -3.523 1.937 -17.990 1.00 13.56 H

ATOM 33 HD2 TYR A 93 -1.182 5.658 -19.117 1.00 10.79 H

ATOM 34 HE2 TYR A 93 -2.100 3.746 -17.883 1.00 11.29 H

ATOM 35 HCAP TYR A 93 -1.118 6.114 -22.294 1.00 10.19 H

ATOM 36 CB ASP A 95 4.008 5.020 -25.494 1.00 12.04 C

ATOM 37 CG ASP A 95 4.414 3.731 -26.269 1.00 15.21 C

ATOM 38 OD1 ASP A 95 4.174 3.524 -27.480 1.00 14.84 O

ATOM 39 OD2 ASP A 95 5.055 2.917 -25.516 1.00 13.45 O1-

ATOM 40 1HB ASP A 95 3.522 4.682 -24.578 1.00 12.18 H

ATOM 41 2HB ASP A 95 4.936 5.474 -25.177 1.00 11.99 H

ATOM 42 HCAP ASP A 95 3.424 5.861 -25.902 1.00 12.04 H

ATOM 43 CB TRP A 96 -0.914 3.625 -26.448 1.00 8.38 C

ATOM 44 CG TRP A 96 -0.350 2.411 -25.770 1.00 9.09 C

ATOM 45 CD1 TRP A 96 0.809 1.702 -25.995 1.00 10.02 C

ATOM 46 NE1 TRP A 96 0.857 0.587 -25.175 1.00 10.92 N

ATOM 47 CE2 TRP A 96 -0.273 0.560 -24.396 1.00 9.86 C

ATOM 48 CD2 TRP A 96 -1.045 1.709 -24.731 1.00 9.07 C

ATOM 49 CE3 TRP A 96 -2.220 1.986 -24.011 1.00 10.17 C

ATOM 50 CZ3 TRP A 96 -2.615 1.111 -23.009 1.00 10.92 C

ATOM 51 CZ2 TRP A 96 -0.693 -0.348 -23.415 1.00 9.83 C

ATOM 52 CH2 TRP A 96 -1.874 -0.063 -22.740 1.00 11.14 C

ATOM 53 1HB TRP A 96 -1.755 3.325 -27.083 1.00 8.44 H

ATOM 54 2HB TRP A 96 -1.322 4.319 -25.702 1.00 8.49 H

ATOM 55 HD1 TRP A 96 1.629 1.913 -26.667 1.00 9.93 H

ATOM 56 HE1 TRP A 96 1.570 -0.140 -25.207 1.00 10.38 H

ATOM 57 HE3 TRP A 96 -2.796 2.884 -24.212 1.00 10.04 H

ATOM 58 HZ3 TRP A 96 -3.497 1.335 -22.422 1.00 10.40 H

ATOM 59 HZ2 TRP A 96 -0.109 -1.232 -23.186 1.00 9.88 H

ATOM 60 HH2 TRP A 96 -2.231 -0.762 -21.987 1.00 10.54 H

ATOM 61 HCAP TRP A 96 -0.208 4.195 -27.065 1.00 8.38 H

ATOM 62 CB THR A 99 3.184 4.019 -30.945 1.00 10.49 C

ATOM 63 OG1 THR A 99 2.503 3.444 -29.840 1.00 11.23 O

ATOM 64 CG2 THR A 99 4.450 4.803 -30.567 1.00 12.29 C

ATOM 65 HB THR A 99 3.487 3.195 -31.605 1.00 10.41 H

ATOM 66 HG1 THR A 99 2.969 3.710 -29.018 1.00 11.16 H

ATOM 67 1HG2 THR A 99 4.929 5.242 -31.448 1.00 11.97 H

ATOM 68 2HG2 THR A 99 5.174 4.146 -30.082 1.00 12.12 H

ATOM 69 3HG2 THR A 99 4.238 5.614 -29.865 1.00 11.90 H

ATOM 70 HCAP THR A 99 2.527 4.654 -31.569 1.00 10.49 H

ATOM 71 CB THR A 100 -2.242 2.916 -31.553 1.00 9.31 C

ATOM 72 OG1 THR A 100 -2.882 3.862 -30.734 1.00 10.41 O

ATOM 73 CG2 THR A 100 -1.652 1.794 -30.693 1.00 10.25 C

ATOM 74 HB THR A 100 -3.038 2.484 -32.165 1.00 9.41 H

ATOM 75 HG1 THR A 100 -2.218 4.337 -30.188 1.00 10.37 H

ATOM 76 1HG2 THR A 100 -1.247 0.979 -31.300 1.00 10.05 H

ATOM 77 2HG2 THR A 100 -2.446 1.383 -30.065 1.00 10.02 H

ATOM 78 3HG2 THR A 100 -0.855 2.161 -30.035 1.00 10.16 H

ATOM 79 HCAP THR A 100 -1.474 3.288 -32.264 1.00 9.31 H

ATOM 80 CB LEU A 103 0.696 0.936 -35.861 1.00 9.38 C

ATOM 81 CG LEU A 103 1.976 0.623 -35.052 1.00 10.24 C

ATOM 82 CD1 LEU A 103 1.599 0.085 -33.665 1.00 10.75 C

ATOM 83 CD2 LEU A 103 2.868 -0.377 -35.792 1.00 10.59 C

ATOM 84 1HB LEU A 103 0.285 -0.006 -36.250 1.00 9.28 H

ATOM 85 2HB LEU A 103 -0.067 1.371 -35.204 1.00 9.65 H

ATOM 86 HG LEU A 103 2.545 1.547 -34.892 1.00 10.22 H

ATOM 87 1HD1 LEU A 103 1.064 0.840 -33.082 1.00 10.68 H

ATOM 88 2HD1 LEU A 103 2.495 -0.210 -33.104 1.00 10.72 H

ATOM 89 3HD1 LEU A 103 0.957 -0.801 -33.744 1.00 10.65 H

ATOM 90 1HD2 LEU A 103 2.285 -1.261 -36.074 1.00 10.58 H

ATOM 91 2HD2 LEU A 103 3.697 -0.730 -35.172 1.00 10.53 H

ATOM 92 3HD2 LEU A 103 3.296 0.047 -36.707 1.00 10.50 H

ATOM 93 HCAP LEU A 103 0.827 1.646 -36.694 1.00 9.38 H

ATOM 94 CB MET A 128 -9.362 3.265 -28.599 1.00 9.00 C

ATOM 95 CG MET A 128 -8.875 1.907 -28.082 1.00 9.49 C

ATOM 96 SD MET A 128 -8.109 0.998 -29.481 1.00 10.90 S

ATOM 97 CE MET A 128 -6.434 1.710 -29.478 1.00 10.59 C

ATOM 98 1HB MET A 128 -8.560 3.664 -29.199 1.00 9.19 H

ATOM 99 2HB MET A 128 -10.189 3.107 -29.293 1.00 8.94 H

ATOM 100 1HG MET A 128 -9.683 1.286 -27.702 1.00 9.76 H

ATOM 101 2HG MET A 128 -8.125 1.965 -27.288 1.00 9.62 H

ATOM 102 1HE MET A 128 -5.904 1.281 -30.331 1.00 10.70 H

ATOM 103 2HE MET A 128 -6.450 2.797 -29.590 1.00 10.57 H

ATOM 104 3HE MET A 128 -5.901 1.441 -28.565 1.00 10.64 H

ATOM 105 HCAP MET A 128 -9.674 4.054 -27.895 1.00 9.00 H

ATOM 106 CB ILE A 129 -5.306 5.994 -26.644 1.00 9.62 C

ATOM 107 CG2 ILE A 129 -4.169 6.395 -25.672 1.00 10.13 C

ATOM 108 CG1 ILE A 129 -4.768 4.995 -27.701 1.00 9.93 C

ATOM 109 CD1 ILE A 129 -4.429 3.585 -27.208 1.00 11.01 C

ATOM 110 HB ILE A 129 -5.619 6.895 -27.187 1.00 9.47 H

ATOM 111 1HG2 ILE A 129 -4.357 7.334 -25.140 1.00 9.89 H

ATOM 112 2HG2 ILE A 129 -3.247 6.535 -26.235 1.00 10.16 H

ATOM 113 3HG2 ILE A 129 -3.966 5.624 -24.920 1.00 10.11 H

ATOM 114 1HG1 ILE A 129 -3.863 5.440 -28.129 1.00 9.96 H

ATOM 115 2HG1 ILE A 129 -5.451 4.906 -28.547 1.00 9.88 H

ATOM 116 HD1 ILE A 129 -3.783 3.601 -26.326 1.00 10.80 H

ATOM 117 HD2 ILE A 129 -3.911 3.035 -27.999 1.00 10.78 H

ATOM 118 HD3 ILE A 129 -5.324 3.010 -26.947 1.00 10.79 H

ATOM 119 HCAP ILE A 129 -6.213 5.611 -26.128 1.00 9.62 H

ATOM 120 CB TRP A 148 -12.529 -1.185 -19.604 1.00 11.57 C

ATOM 121 CG TRP A 148 -12.129 -2.482 -20.275 1.00 11.39 C

ATOM 122 CD1 TRP A 148 -10.888 -2.798 -20.770 1.00 12.11 C

ATOM 123 NE1 TRP A 148 -10.924 -4.022 -21.411 1.00 13.19 N

ATOM 124 CE2 TRP A 148 -12.190 -4.554 -21.294 1.00 13.62 C

ATOM 125 CD2 TRP A 148 -12.982 -3.611 -20.586 1.00 11.93 C

ATOM 126 CE3 TRP A 148 -14.330 -3.923 -20.325 1.00 15.15 C

ATOM 127 CZ3 TRP A 148 -14.839 -5.138 -20.767 1.00 17.56 C

ATOM 128 CZ2 TRP A 148 -12.695 -5.780 -21.738 1.00 16.01 C

ATOM 129 CH2 TRP A 148 -14.030 -6.059 -21.467 1.00 17.33 C

ATOM 130 1HB TRP A 148 -13.373 -1.366 -18.937 1.00 11.59 H

ATOM 131 2HB TRP A 148 -11.714 -0.802 -18.985 1.00 11.56 H

ATOM 132 HD1 TRP A 148 -9.973 -2.227 -20.745 1.00 12.33 H

ATOM 133 HE1 TRP A 148 -10.103 -4.584 -21.606 1.00 12.74 H

ATOM 134 HE3 TRP A 148 -14.965 -3.225 -19.787 1.00 14.70 H

ATOM 135 HZ3 TRP A 148 -15.879 -5.387 -20.572 1.00 16.70 H

ATOM 136 HZ2 TRP A 148 -12.064 -6.487 -22.268 1.00 15.56 H

ATOM 137 HH2 TRP A 148 -14.458 -7.002 -21.798 1.00 16.25 H

ATOM 138 HCAP TRP A 148 -12.808 -0.350 -20.273 1.00 11.57 H

ATOM 139 CB SER A 151 -11.549 1.949 -24.181 1.00 9.86 C

ATOM 140 OG SER A 151 -10.248 1.847 -24.728 1.00 10.47 O

ATOM 141 1HB SER A 151 -11.688 1.082 -23.532 1.00 9.93 H

ATOM 142 2HB SER A 151 -11.674 2.834 -23.553 1.00 10.01 H

ATOM 143 HG1 SER A 151 -9.882 2.740 -24.877 1.00 10.52 H

ATOM 144 HCAP SER A 151 -12.358 1.941 -24.932 1.00 9.86 H

ATOM 145 CB THR A 152 -13.408 -2.893 -25.362 1.00 10.97 C

ATOM 146 OG1 THR A 152 -12.412 -3.000 -24.340 1.00 11.95 O

ATOM 147 CG2 THR A 152 -13.458 -4.277 -26.011 1.00 13.27 C

ATOM 148 HB THR A 152 -14.390 -2.670 -24.925 1.00 11.13 H

ATOM 149 HG1 THR A 152 -12.595 -2.317 -23.666 1.00 11.95 H

ATOM 150 1HG2 THR A 152 -14.266 -4.379 -26.744 1.00 12.88 H

ATOM 151 2HG2 THR A 152 -13.621 -5.008 -25.214 1.00 12.81 H

ATOM 152 3HG2 THR A 152 -12.509 -4.517 -26.502 1.00 12.97 H

ATOM 153 HCAP THR A 152 -13.149 -2.055 -26.043 1.00 10.97 H

ATOM 154 CB MET A 155 -10.336 -1.625 -29.626 1.00 10.21 C

ATOM 155 CG MET A 155 -9.129 -2.420 -30.148 1.00 11.14 C

ATOM 156 SD MET A 155 -8.690 -3.828 -29.079 1.00 11.57 S

ATOM 157 CE MET A 155 -10.188 -4.861 -29.281 1.00 12.74 C

ATOM 158 1HB MET A 155 -11.082 -2.296 -29.197 1.00 10.18 H

ATOM 159 2HB MET A 155 -10.014 -0.956 -28.826 1.00 10.42 H

ATOM 160 1HG MET A 155 -8.239 -1.787 -30.174 1.00 11.23 H

ATOM 161 2HG MET A 155 -9.261 -2.822 -31.155 1.00 11.12 H

ATOM 162 1HE MET A 155 -10.067 -5.741 -28.647 1.00 12.51 H

ATOM 163 2HE MET A 155 -11.089 -4.333 -28.962 1.00 12.56 H

ATOM 164 3HE MET A 155 -10.301 -5.202 -30.311 1.00 12.56 H

ATOM 165 HCAP MET A 155 -10.837 -1.014 -30.392 1.00 10.21 H

ATOM 166 CB TRP A 192 -4.824 -4.457 -31.407 1.00 9.58 C

ATOM 167 CG TRP A 192 -3.723 -3.671 -32.079 1.00 10.20 C

ATOM 168 CD1 TRP A 192 -2.430 -4.020 -32.400 1.00 10.86 C

ATOM 169 NE1 TRP A 192 -1.784 -2.979 -33.040 1.00 11.18 N

ATOM 170 CE2 TRP A 192 -2.655 -1.915 -33.147 1.00 10.92 C

ATOM 171 CD2 TRP A 192 -3.885 -2.321 -32.553 1.00 10.74 C

ATOM 172 CE3 TRP A 192 -4.978 -1.436 -32.554 1.00 10.97 C

ATOM 173 CZ3 TRP A 192 -4.818 -0.175 -33.110 1.00 11.88 C

ATOM 174 CZ2 TRP A 192 -2.489 -0.639 -33.701 1.00 11.97 C

ATOM 175 CH2 TRP A 192 -3.582 0.220 -33.663 1.00 11.71 C

ATOM 176 1HB TRP A 192 -5.484 -3.749 -30.892 1.00 9.51 H

ATOM 177 2HB TRP A 192 -5.457 -4.995 -32.118 1.00 9.67 H

ATOM 178 HD1 TRP A 192 -1.891 -4.940 -32.229 1.00 10.59 H

ATOM 179 HE1 TRP A 192 -0.847 -3.041 -33.434 1.00 10.96 H

ATOM 180 HE3 TRP A 192 -5.934 -1.738 -32.136 1.00 10.96 H

ATOM 181 HZ3 TRP A 192 -5.650 0.519 -33.120 1.00 11.78 H

ATOM 182 HZ2 TRP A 192 -1.549 -0.330 -34.145 1.00 11.76 H

ATOM 183 HH2 TRP A 192 -3.480 1.224 -34.054 1.00 11.52 H

ATOM 184 HCAP TRP A 192 -4.453 -5.169 -30.656 1.00 9.58 H

ATOM 185 CB TYR A 195 -4.167 -3.967 -25.510 1.00 9.35 C

ATOM 186 CG TYR A 195 -2.749 -3.870 -26.024 1.00 8.83 C

ATOM 187 CD1 TYR A 195 -2.357 -4.582 -27.160 1.00 9.54 C

ATOM 188 CE1 TYR A 195 -1.087 -4.479 -27.706 1.00 9.39 C

ATOM 189 CZ TYR A 195 -0.143 -3.648 -27.092 1.00 8.71 C

ATOM 190 OH TYR A 195 1.067 -3.524 -27.682 1.00 9.91 O

ATOM 191 CD2 TYR A 195 -1.792 -3.052 -25.419 1.00 9.16 C

ATOM 192 CE2 TYR A 195 -0.496 -2.958 -25.922 1.00 9.21 C

ATOM 193 1HB TYR A 195 -4.353 -3.145 -24.811 1.00 9.16 H

ATOM 194 2HB TYR A 195 -4.865 -3.837 -26.336 1.00 9.38 H

ATOM 195 HD1 TYR A 195 -3.079 -5.235 -27.619 1.00 9.41 H

ATOM 196 HE1 TYR A 195 -0.810 -5.038 -28.594 1.00 9.25 H

ATOM 197 HH TYR A 195 1.652 -2.957 -27.112 1.00 9.89 H

ATOM 198 HD2 TYR A 195 -2.058 -2.449 -24.557 1.00 9.04 H

ATOM 199 HE2 TYR A 195 0.240 -2.325 -25.444 1.00 9.12 H

ATOM 200 HCAP TYR A 195 -4.406 -4.922 -25.010 1.00 9.35 H

ATOM 201 CB TRP A 199 -6.574 -3.409 -19.769 1.00 10.80 C

ATOM 202 CG TRP A 199 -6.635 -2.046 -19.142 1.00 11.22 C

ATOM 203 CD1 TRP A 199 -5.645 -1.101 -19.235 1.00 10.88 C

ATOM 204 NE1 TRP A 199 -6.060 0.102 -18.702 1.00 11.68 N

ATOM 205 CE2 TRP A 199 -7.352 -0.049 -18.250 1.00 11.95 C

ATOM 206 CD2 TRP A 199 -7.737 -1.400 -18.476 1.00 10.91 C

ATOM 207 CE3 TRP A 199 -8.989 -1.831 -17.999 1.00 11.76 C

ATOM 208 CZ3 TRP A 199 -9.808 -0.934 -17.323 1.00 12.13 C

ATOM 209 CZ2 TRP A 199 -8.203 0.871 -17.630 1.00 13.05 C

ATOM 210 CH2 TRP A 199 -9.427 0.414 -17.160 1.00 13.29 C

ATOM 211 1HB TRP A 199 -7.542 -3.666 -20.212 1.00 10.79 H

ATOM 212 2HB TRP A 199 -5.865 -3.368 -20.603 1.00 10.88 H

ATOM 213 HD1 TRP A 199 -4.664 -1.186 -19.678 1.00 11.18 H

ATOM 214 HE1 TRP A 199 -5.525 0.966 -18.752 1.00 11.36 H

ATOM 215 HE3 TRP A 199 -9.299 -2.861 -18.132 1.00 11.63 H

ATOM 216 HZ3 TRP A 199 -10.755 -1.269 -16.913 1.00 11.98 H

ATOM 217 HZ2 TRP A 199 -7.901 1.903 -17.540 1.00 12.54 H

ATOM 218 HH2 TRP A 199 -10.103 1.097 -16.654 1.00 12.83 H

ATOM 219 HCAP TRP A 199 -6.243 -4.250 -19.140 1.00 10.80 H

ATOM 220 CB ASP A 222 4.776 -2.696 -26.177 1.00 9.27 C

ATOM 221 CG ASP A 222 3.597 -1.993 -25.496 1.00 11.03 C

ATOM 222 OD1 ASP A 222 2.442 -1.951 -25.998 1.00 11.31 O

ATOM 223 OD2 ASP A 222 3.930 -1.338 -24.463 1.00 13.10 O1-

ATOM 224 1HB ASP A 222 5.332 -1.877 -26.646 1.00 9.46 H

ATOM 225 2HB ASP A 222 5.423 -3.143 -25.419 1.00 9.40 H

ATOM 226 HCAP ASP A 222 4.527 -3.457 -26.924 1.00 9.27 H

ATOM 227 CB ALA A 225 2.152 -4.420 -31.194 1.00 13.00 C

ATOM 228 1HB ALA A 225 1.428 -3.838 -31.768 1.00 12.74 H

ATOM 229 2HB ALA A 225 1.629 -5.229 -30.682 1.00 12.84 H

ATOM 230 3HB ALA A 225 2.597 -3.786 -30.424 1.00 12.85 H

ATOM 231 HCAP ALA A 225 2.930 -4.845 -31.844 1.00 13.00 H

ATOM 232 CB LYS A 226 6.474 -1.556 -31.603 1.00 11.71 C

ATOM 233 CG LYS A 226 5.272 -0.843 -30.967 1.00 11.29 C

ATOM 234 CD LYS A 226 5.645 0.256 -29.981 1.00 12.10 C

ATOM 235 CE LYS A 226 4.424 1.100 -29.635 1.00 13.89 C

ATOM 236 NZ LYS A 226 3.411 0.356 -28.889 1.00 15.97 N1+

ATOM 237 1HB LYS A 226 7.208 -0.828 -31.968 1.00 11.53 H

ATOM 238 2HB LYS A 226 7.005 -2.163 -30.865 1.00 11.56 H

ATOM 239 1HG LYS A 226 4.619 -1.562 -30.466 1.00 11.51 H

ATOM 240 2HG LYS A 226 4.646 -0.398 -31.751 1.00 11.33 H

ATOM 241 1HD LYS A 226 6.399 0.923 -30.416 1.00 12.02 H

ATOM 242 2HD LYS A 226 6.075 -0.154 -29.063 1.00 12.25 H

ATOM 243 1HE LYS A 226 3.956 1.482 -30.543 1.00 13.48 H

ATOM 244 2HE LYS A 226 4.676 1.954 -29.005 1.00 13.77 H

ATOM 245 1HZ LYS A 226 3.624 0.323 -27.868 1.00 15.85 H

ATOM 246 HCAP LYS A 226 6.130 -2.180 -32.442 1.00 11.71 H

TER 247 LYS A 226

HETATM 248 C1 DMP r 300 -6.674 -0.469 -23.057 1.00 9.37 C

HETATM 249 C2 DMP r 300 -7.434 -0.047 -21.966 1.00 9.74 C

HETATM 250 C3 DMP r 300 -8.828 -0.045 -22.011 1.00 9.20 C

HETATM 251 C4 DMP r 300 -9.464 -0.508 -23.154 1.00 9.43 C

HETATM 252 C5 DMP r 300 -8.736 -0.826 -24.306 1.00 9.51 C

HETATM 253 C6 DMP r 300 -7.323 -0.743 -24.296 1.00 9.12 C

HETATM 254 C7 DMP r 300 -6.622 -0.961 -25.564 1.00 9.30 C

HETATM 255 C8 DMP r 300 -5.405 -0.508 -25.955 1.00 9.01 C

HETATM 256 C9 DMP r 300 -4.741 -0.841 -27.198 1.00 9.11 C

HETATM 257 C10 DMP r 300 -3.406 -0.530 -27.281 1.00 8.74 C

HETATM 258 C11 DMP r 300 -2.477 -0.888 -28.301 1.00 9.24 C

HETATM 259 C12 DMP r 300 -1.138 -0.673 -28.104 1.00 9.25 C

HETATM 260 C13 DMP r 300 -0.066 -0.865 -29.030 1.00 9.08 C

HETATM 261 C14 DMP r 300 1.191 -0.473 -28.593 1.00 9.47 C

HETATM 262 C15 DMP r 300 2.283 -0.091 -29.392 1.00 10.61 C

HETATM 263 C16 DMP r 300 -5.231 -0.829 -22.795 1.00 9.68 C

HETATM 264 C18 DMP r 300 -9.520 -1.219 -25.527 1.00 9.05 C

HETATM 265 C19 DMP r 300 -5.477 -1.589 -28.278 1.00 9.19 C

HETATM 266 C20 DMP r 300 -0.299 -1.495 -30.371 1.00 9.42 C

HETATM 267 H21 DMP r 300 -6.932 0.216 -21.044 1.00 9.59 H

HETATM 268 H31 DMP r 300 -9.397 0.261 -21.137 1.00 9.30 H

HETATM 269 H41 DMP r 300 -10.543 -0.608 -23.179 1.00 9.51 H

HETATM 270 H71 DMP r 300 -7.184 -1.508 -26.311 1.00 9.25 H

HETATM 271 H81 DMP r 300 -4.823 0.117 -25.288 1.00 9.05 H

HETATM 272 H101 DMP r 300 -2.968 -0.020 -26.426 1.00 8.82 H

HETATM 273 H111 DMP r 300 -2.830 -1.354 -29.216 1.00 9.21 H

HETATM 274 H121 DMP r 300 -0.852 -0.228 -27.157 1.00 9.21 H

HETATM 275 H141 DMP r 300 1.304 -0.282 -27.536 1.00 9.51 H

HETATM 276 H15 DMP r 300 2.190 -0.040 -30.474 1.00 10.12 H

HETATM 277 H161 DMP r 300 -5.128 -1.923 -22.789 1.00 9.62 H

HETATM 278 H162 DMP r 300 -4.932 -0.471 -21.808 1.00 9.63 H

HETATM 279 H163 DMP r 300 -4.513 -0.451 -23.512 1.00 9.66 H

HETATM 280 H181 DMP r 300 -9.035 -1.970 -26.156 1.00 9.08 H

HETATM 281 H182 DMP r 300 -9.728 -0.343 -26.147 1.00 9.16 H

HETATM 282 H183 DMP r 300 -10.465 -1.643 -25.191 1.00 9.16 H

HETATM 283 H191 DMP r 300 -5.660 -2.629 -27.987 1.00 9.14 H

HETATM 284 H192 DMP r 300 -4.917 -1.603 -29.215 1.00 9.24 H

HETATM 285 H193 DMP r 300 -6.451 -1.129 -28.472 1.00 9.19 H

HETATM 286 H201 DMP r 300 0.502 -1.278 -31.082 1.00 9.33 H

HETATM 287 H202 DMP r 300 -1.251 -1.205 -30.816 1.00 9.34 H

HETATM 288 H203 DMP r 300 -0.317 -2.582 -30.226 1.00 9.44 H

HETATM 289 O HOH o 401 3.277 1.961 -23.791 1.00 15.83 O

HETATM 290 H1 HOH o 401 2.491 2.439 -24.101 1.00 0.00 H

HETATM 291 H2 HOH o 401 4.038 2.328 -24.315 1.00 0.00 H

HETATM 292 O HOH o 402 4.718 0.490 -26.517 1.00 23.77 O

HETATM 293 H1 HOH o 402 4.433 0.120 -25.660 1.00 0.00 H

HETATM 294 H2 HOH o 402 4.949 1.438 -26.294 1.00 0.00 H

HETATM 295 O HOH o 406 2.519 -0.283 -22.590 1.00 15.78 O

HETATM 296 H1 HOH o 406 2.895 -0.846 -23.323 1.00 0.00 H

HETATM 297 H2 HOH o 406 2.832 0.611 -22.889 1.00 0.00 H

ENDMDL

END
